# Global Analysis of Fission Yeast Mating Genes Reveals New Autophagy Factors

**DOI:** 10.1371/journal.pgen.1003715

**Published:** 2013-08-08

**Authors:** Ling-Ling Sun, Ming Li, Fang Suo, Xiao-Man Liu, En-Zhi Shen, Bing Yang, Meng-Qiu Dong, Wan-Zhong He, Li-Lin Du

**Affiliations:** National Institute of Biological Sciences, Beijing, China; Duke University Medical Center, United States of America

## Abstract

Macroautophagy (autophagy) is crucial for cell survival during starvation and plays important roles in animal development and human diseases. Molecular understanding of autophagy has mainly come from the budding yeast *Saccharomyces cerevisiae*, and it remains unclear to what extent the mechanisms are the same in other organisms. Here, through screening the mating phenotype of a genome-wide deletion collection of the fission yeast *Schizosaccharomyces pombe*, we obtained a comprehensive catalog of autophagy genes in this highly tractable organism, including genes encoding three heretofore unidentified core Atg proteins, Atg10, Atg14, and Atg16, and two novel factors, Ctl1 and Fsc1. We systematically examined the subcellular localization of fission yeast autophagy factors for the first time and characterized the phenotypes of their mutants, thereby uncovering both similarities and differences between the two yeasts. Unlike budding yeast, all three Atg18/WIPI proteins in fission yeast are essential for autophagy, and we found that they play different roles, with Atg18a uniquely required for the targeting of the Atg12–Atg5·Atg16 complex. Our investigation of the two novel factors revealed unforeseen autophagy mechanisms. The choline transporter-like protein Ctl1 interacts with Atg9 and is required for autophagosome formation. The fasciclin domain protein Fsc1 localizes to the vacuole membrane and is required for autophagosome-vacuole fusion but not other vacuolar fusion events. Our study sheds new light on the evolutionary diversity of the autophagy machinery and establishes the fission yeast as a useful model for dissecting the mechanisms of autophagy.

## Author Summary

Autophagy is a eukaryotic cellular process that transports cytoplasmic contents into lysosomes/vacuoles for degradation. It has been linked to multiple human diseases, including cancer and neurodegenerative disorders. The molecular machinery of autophagy was first identified and has been best characterized in the budding yeast *Saccharomyces cerevisiae*, but little is known about the autophagy machinery in another important unicellular model organism, the fission yeast *Schizosaccharomyces pombe*. In this study, we performed an unbiased and comprehensive screening of the fission yeast autophagy genes by profiling the mating phenotypes of nearly 3000 deletion strains. Following up on the screening results, we systematically characterized both previously known and newly identified fission yeast autophagy factors by examining their localization and the phenotype of their mutants. Our analysis increased the number of experimentally defined fission yeast autophagy factors from 14 to 23, including two novel factors that act in ways different from all previously known autophagy proteins. Together, our data reveal unexpected evolutionary divergence of autophagy mechanisms and establish a new model system for unraveling the molecular details of the autophagy process.

## Introduction

Macroautophagy (hereafter autophagy) is a catabolic pathway that transports cytoplasmic materials into a degradative organelle, the vacuole or lysosome. This self-digestion process is upregulated during starvation, when cells have to rely on the turnover of intracellular substances to provide the building blocks for synthesizing new macromolecules [1]. Autophagy is critically important for the survival of unicellular organisms such as yeasts, whose cells are directly exposed to a fluctuating environment [2], [3]. In recent years, diverse roles of autophagy in the development and health of multicellular organisms have also been uncovered [4], [5].

Molecular understanding of autophagy began with the identification of autophagy-related (*ATG*) genes in *S. cerevisiae*, which remains the organism where the autophagy machinery has been best characterized [6]–[8]. The Atg proteins required for all autophagy-related pathways are referred to as the core Atg proteins, and most of them are involved in the generation of a double membrane-enclosed transport vehicle called autophagosome. Two protein complexes are important for initiating the autophagosome formation process. One complex consists of Atg1 kinase and its associated proteins. The other is the phosphatidylinositol 3-kinase (PI3K) complex composed of Vps34, Vps15, Atg6, and Atg14, which generates phosphatidylinositol 3-phosphate (PI3P) at the sites where autophagosomes are assembled. PI3P is recognized by Atg18 (homolog of mammalian WIPI proteins), which together with Atg2, regulates the retrograde trafficking of Atg9, the only core Atg protein with transmembrane domains. The expansion of the autophagosome precursor, called isolation membrane or phagophore, requires the conjugation of a ubiquitin-like protein Atg8 to phosphatidylethanolamine. Factors involved in this conjugation include the Atg8 processing enzyme Atg4, the E1 enzyme Atg7, the E2 enzyme Atg3, and the E3-like complex Atg12–Atg5·Atg16. Atg12 is another ubiquitin-like protein whose conjugation to Atg5 requires the E2 enzyme Atg10.

Many *ATG* genes in *S. cerevisiae* have readily recognizable homologs in other eukaryotes, indicating that autophagy is an ancient and conserved pathway. On the other hand, differences in the autophagy machinery between *S. cerevisiae* and other organisms have also been documented and it has been argued that studying additional model organisms will help us better understand the evolution and mechanisms of autophagy [9], [10]. The fission yeast *S. pombe* is evolutionarily very distant from *S. cerevisiae*. Molecular clock studies estimated that these two species diverged more than 500 million years ago [11]. The existence of a homolog of mammalian Atg101 in *S. pombe* but not *S. cerevisiae* underscores the potential value of *S. pombe* for studying the divergences of autophagy mechanisms [12]. However, no screen for autophagy genes has been conducted in *S. pombe*, and published works on fission yeast autophagy have been limited to a subset of close homologs of budding yeast *ATG* genes [3], [13].

In this study, through unbiased genome-wide screening, we discovered new autophagy factors in *S. pombe*. Characterization of these and other autophagy factors in fission yeast demonstrated the utility of *S. pombe* in uncovering novel autophagy mechanisms.

## Results

### A genome-wide analysis of mating genes in fission yeast

Unlike *S. cerevisiae*, haploid *S. pombe* cells of opposite mating types (*h^−^* or *h^+^*) do not mate on the standard rich medium. Instead, mating is most efficiently triggered by nitrogen starvation, and immediately followed by meiosis and sporulation [14], [15]. From classical genetic screens, about 20 mating genes have been identified in *S. pombe*, which are called *ste* (for sterile) or *ral* (for *ras*-like) genes [16 and references therein]. According to PomBase [17], 15 *ste* and *ral* genes have been cloned thus far. The classical screens are far from saturating, as a few dozen additional genes, uncovered through other means, have been implicated in mating [15]. The mating defect of autophagy mutants, which is attributed to an inability to supply enough nitrogen intracellularly, was only discovered during a focused study on these mutants [3].

**Figure 1:**
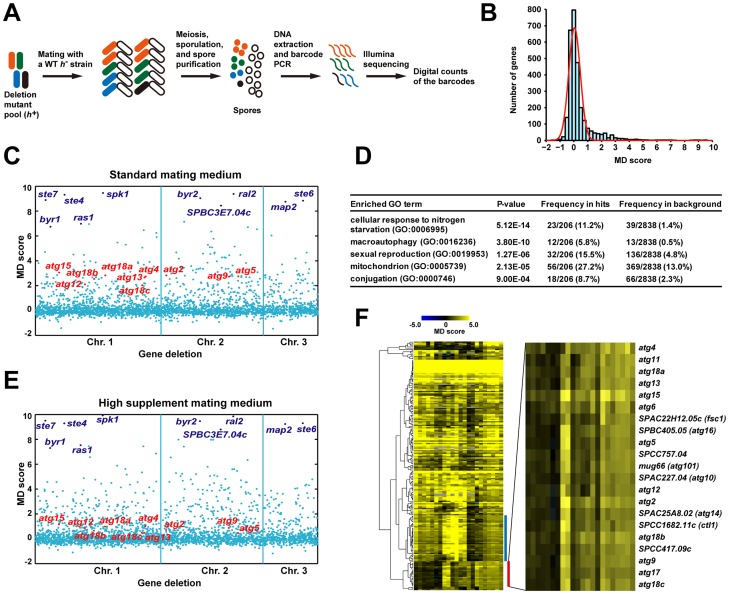
Barcode sequencing-based screens of mating phenotype. (A) Schematic of the screening procedure. (B) A histogram of the mating defect (MD) scores from the screen 0428_YES_SPA-45s conducted under standard mating conditions. The red line represents a fitted normal distribution. (C) A scatter plot of the MD scores from the screen 0428_YES_SPA-45s. The genes are ordered according to their chromosomal positions. The 10 genes with the highest average MD scores under standard conditions are highlighted in dark blue. The 10 genes known to be required for starvation-induced autophagy are highlighted in red. (D) Gene Ontology (GO) term enrichment analysis of the screen hits obtained under standard conditions. (E) A scatter plot of the MD scores from the screen 0428_YES_SPA-200s. Genes are highlighted as in C. (F) Hierarchical clustering analysis of the MD scores from the 22 screens. For a detailed view of the heat map, see Figure S2. Blue bar denotes the cluster enriched for mitochondrial protein-coding genes. Red bar denotes the autophagy gene cluster, whose close-up view is shown at right.

With an aim of identifying new autophagy genes, we screened the mating phenotype of a fission yeast deletion collection [18], using a barcode sequencing technology we have developed [19]. In our screening procedure (Figure 1A), we mixed a pool of haploid deletion strains, which are *h^+^*, with an equal amount of wild-type (WT) *h^−^* cells on solid mating media. After 4 days of incubation, we isolated the spores from the mating mixtures. Genomic DNA was extracted from both the input mutant pool and the spores. Barcodes associated with the deletions were amplified by PCR and sequenced. For each mutant/gene, we calculated a mating defect (MD) score, which is a normalized log2 ratio of barcode sequencing counts in input vs. spores. For mating genes, we expected MD scores higher than 0 because their barcodes should be depleted among the spores. On the other hand, genes involved in meiosis and/or sporulation but not mating should have MD scores close to 0, as their deletions are unlikely to manifest a phenotype in the heterozygous diploid state.

Under the standard mating conditions (pregrowth in liquid YES medium, and mating on the SPA solid medium supplemented with 45 mg/l of leucine, uracil, and adenine) [20], we obtained MD scores for more than 2800 mutants, representing about 80% of the non-essential *S. pombe* genes. The distribution of MD scores largely conforms to a normal distribution centered at 0, except for a long right tail, which represents mating-defective mutants with higher than usual MD scores (Figure 1B and 1C). We repeated the screen twice, and identified the mating-defective mutants as the ones passing a false discovery rate (FDR) cutoff of 0.1 in all three screens (Figure S1). Using this stringent criterion, a total of 206 deletion mutants were found to be mating-defective, to different extents, under the standard mating conditions (Table S1).

The mutants of 9 *ste* and *ral* genes (*byr1/ste1*, *ste4*, *ras1/ste5*, *ste6*, *ste7*, *byr2/ste8*, *ste20*, *ral2*, *scd2/ral3*) were among the deletion strains screened. It was satisfying to see all of them scored as mating-defective by our analysis. Moreover, seven of them were among the top 10 hits ranked by the average MD scores (Table S1). It is probably no coincidence that all of these *ste* and *ral* genes are involved in the nutrient sensing or pheromone response pathways, as mutants blocking these signaling pathways are known to have the most severe mating defect [14], [15].

As expected, Gene Ontology (GO) term analysis showed that among the 206 screen hits, genes involved in starvation response, sexual reproduction, and macroautophagy are significantly enriched (Figure 1D). Surprisingly, genes encoding mitochondrial proteins are also heavily enriched, suggesting that mitochondria may play a previously under-appreciated role in mating.

Using barcode sequencing-based analysis, we could recapitulate the finding that the severity of the mating defect of autophagy mutants is influenced by the mating conditions [3]. Raising the concentrations of supplements in the mating medium from 45 mg/l to 200 mg/l led to a significant reduction of the MD scores of autophagy mutants, but did not change those of the top-ranked signaling mutants (compare Figure 1C and 1E). This observation suggests that extending our analysis to alternative mating conditions may help classifying mating genes into different functional categories. Thus, in addition to the three screens performed under the standard mating conditions, we conducted 19 screens under 18 non-standard conditions (Table S2), resulting in a total of 63146 MD score measurements for 2915 mutants (Table S3).

As predicted, hierarchical clustering of the data from the 22 screens revealed conspicuous patterns of phenotypic variations among the mating genes, with many falling into tight clusters (Figure 1F and Figure S2). Two of the most distinct clusters are enriched for mitochondrial protein-coding genes and autophagy genes, respectively. In this study, we focused on the genes in the autophagy cluster, but our extensive phenotyping data should be a useful resource for future investigation on other genes and cellular processes.

### The autophagy cluster contains previously unknown fission yeast autophagy genes

There are 21 genes in the autophagy cluster (Figure 1F), including 10 genes known to be required for starvation-induced autophagy (*atg2*, *atg4*, *atg5*, *atg9*, *atg12*, *atg13*, *atg15*, *atg18a*, *atg18b*, *atg18c*) [3], [13]. These 10 genes represent all known fission yeast autophagy factors detectable by our analysis, as the other 4 characterized autophagy genes did not have MD scores, two due to lack of deletion strains (*atg3* and *atg8*), one due to lack of decoded barcodes (*atg1*), and one due to low barcode read counts (*atg7*). Among the remaining 11 genes in the autophagy cluster, *atg6*, *atg11*, *atg17*, and *atg101* have reported homology to autophagy genes in other organisms [21], but no experimental data on these 4 genes have been published. We hypothesized that these 4 genes, as well as the other 7 genes, which are unnamed and have no reported connections to autophagy, may also function in starvation-induced autophagy.

**Figure 2:**
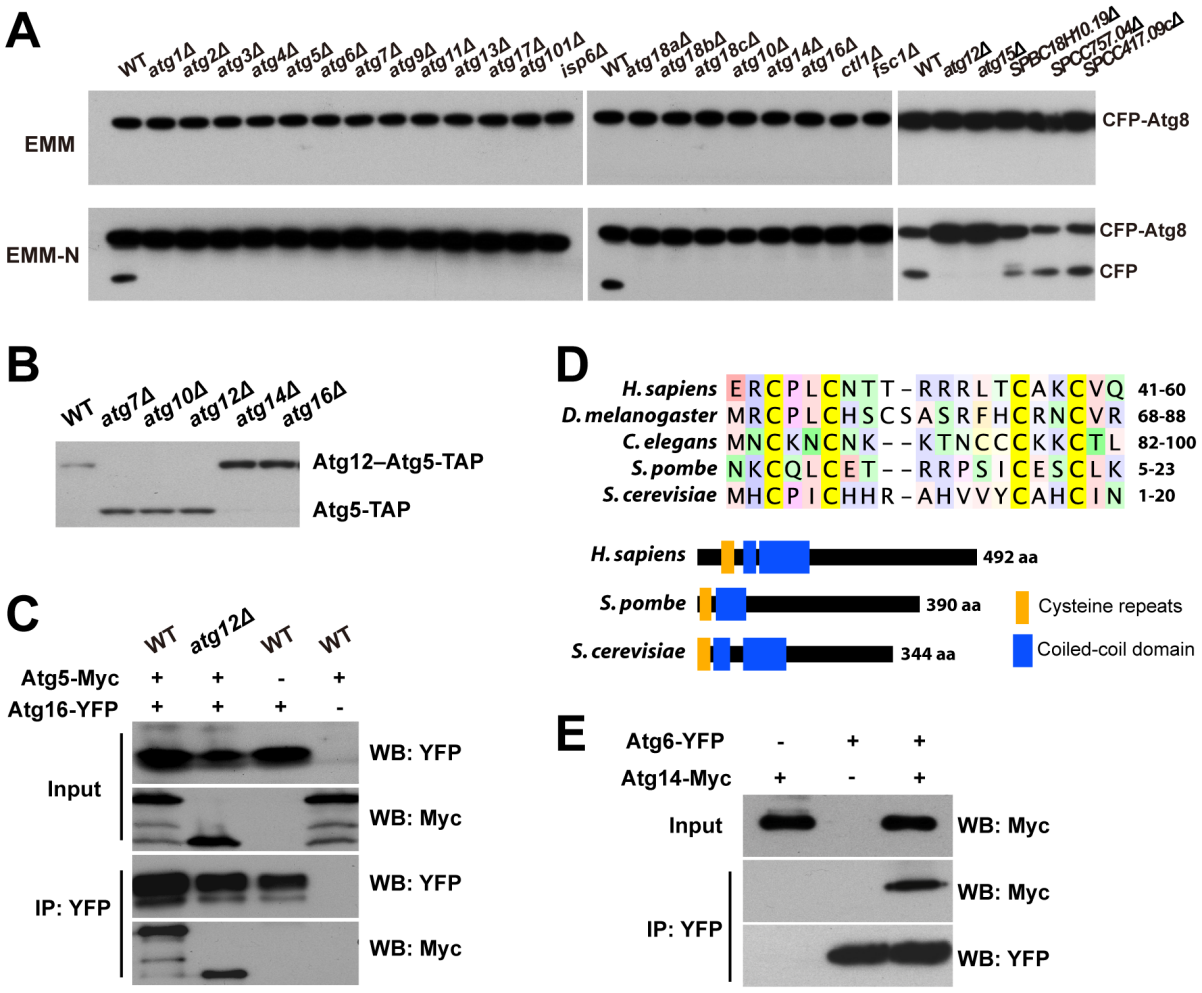
CFP-Atg8 processing defect of autophagy mutants and the identification of Atg10, Atg16, and Atg14. (A) CFP-Atg8 processing assay. Cells were collected before and 8 h after shifting to a nitrogen-free medium (EMM-N). (B) The conjugation of Atg12 to Atg5 requires the *atg10* gene. (C) Atg5-Myc was co-immunoprecipitated with Atg16-YFP in both wild-type and *atg12*Δ cells. Input, 1%; IP, 20%. (D) The “cysteine repeats” region and the domain organization of Atg14 proteins. Coiled-coil domains are predicted as in Figure S4. (E) Atg14-Myc was co-immunoprecipitated with Atg6-YFP. Input, 1%; IP, 20%.

To test this hypothesis, we monitored nitrogen starvation-induced autophagy using the Atg8 fusion protein processing assay [13], [22], [23] (Figure 2A). We constructed a strain expressing from the endogenous promoter an Atg8 protein N-terminally tagged with cyan fluorescent protein (CFP), and then introduced the deletions of the autophagy cluster genes individually into this strain by PCR-based gene targeting. When wild-type (WT) cells expressing CFP-Atg8 were shifted from a growth medium (EMM) to a nitrogen-free medium (EMM-N), immunoblotting analysis showed the appearance of a free CFP band, due to the autophagic delivery of CFP-Atg8 into vacuoles and the subsequent proteolysis that releases the protease-resistant CFP. The processing of CFP-Atg8 was not observed in the 14 mutants known to be defective in autophagy or vacuolar proteolysis (*atg1*, *atg2*, *atg3*, *atg4*, *atg5*, *atg7*, *atg9*, *atg12*, *atg13*, *atg15*, *atg18a*, *atg18b*, *atg18c*, and *isp6*). In addition, we found that *atg6*, *atg11*, *atg17*, and *atg101* are also required for CFP-Atg8 processing, thus providing for the first time evidence that they are required for autophagy. Among the 7 unnamed genes, five are also required for CFP-Atg8 processing. For reasons that will be explained below, we give these five genes the names of *atg10*, *atg14*, *atg16*, *ctl1*, and *fsc1*, respectively. The other two genes (*SPCC757.04* and *SPCC417.09c*), when deleted, had no effect on CFP-Atg8 processing. We suspect that the deletion library strains for these two genes may harbor background mutations that interfere with autophagy. These two genes were not pursued further.

### Identification of *S. pombe* Atg10, Atg16, and Atg14

Three core components of the autophagy machinery, Atg10, Atg16, and Atg14, have not been identified in *S. pombe* [21], [24]. We found that three new autophagy genes uncovered in our screens share distant homology with genes encoding these three Atg proteins in other species.

SPAC227.04 contains a Pfam domain (PF07238) associated with Atg3 and Atg10 proteins. Our sequence homology analysis suggested that SPAC227.04 is more closely related to Atg10 proteins in metazoa and plants than to any Atg3 proteins (Figure S3). If SPAC227.04 is indeed Atg10 in *S. pombe*, removing it should abolish the conjugation of Atg12 to Atg5. In crude extracts made from wild-type cells expressing TAP-tagged Atg5 (Atg5-TAP), we detected by immunoblotting one major band of Atg5-TAP, presumably in the form of Atg12–Atg5 conjugate, as this band disappeared in *atg12*Δ extracts, as well as in *atg7*Δ extracts, which is defective in the E1 enzyme (Figure 2B). A faster-migrating band, likely representing the free form of Atg5, appeared in *atg12*Δ and *atg7*Δ extracts. When *SPAC227.04/atg10* was deleted, only the free form of Atg5 was detected, thus confirming our prediction.

SPBC405.05 is currently annotated as a sequence orphan. We found that it shares homology with *S. cerevisiae* Atg16 in both the N-terminal Atg5-binding domain and the C-terminal coiled-coil domain (Figure S4). Similar to what has been reported for *S. cerevisiae* [25], we found in a co-immunoprecipitation (co-IP) experiment that, *S. pombe* SPBC405.05/Atg16 protein interacts with Atg5 both in the presence and in the absence of Atg12 (Figure 2C).

SPAC25A8.02, also annotated as a sequence orphan, was shown by our PSI-BLAST analysis to be related to metazoan Atg14 proteins. The most conserved sequence feature in Atg14 proteins is a pair of CXXC motifs termed “cysteine repeats” [26]. SPAC25A8.02 contains such a sequence feature, as well as a coiled-coil domain following the cysteine repeats, thus sharing the same domain arrangement with Atg14 proteins in other organisms (Figure 2D). Consistent with our homology analysis, we could co-immunoprecipitate SPAC25A8.02/Atg14 with Atg6, the expected binding partner of Atg14 in a PI3K complex (Figure 2E).

In PomBase, another gene, *SPBC18H10.19*, is currently annotated as *atg14* because of its match to a Pfam domain (PF10186) associated with budding yeast Atg14. This domain is also found in metazoan UVRAG and Atg14 proteins, which are mutually exclusive subunits of Beclin 1-containing PI3K complexes [27]. In budding yeast, the likely counterpart of UVRAG, Vps38, resides in a PI3K complex distinct from the Atg14-containing complex and is dispensable for autophagy [28]. SPBC18H10.19 lacks the N-terminal cysteine repeats typical for the Atg14 proteins. Furthermore, the deletion library strain of *SPBC18H10.19* showed no mating defect in our screens and an independent deletion made in the CFP-Atg8 strain did not block starvation-induced CFP-Atg8 processing (Figure 2A). Thus, we conclude that SPAC25A8.02 is the *S. pombe* Atg14, and SPBC18H10.19 may be the fission yeast equivalent of metazoan UVRAG and budding yeast Vps38.

### Subcellular localization of *S. pombe* Atg proteins

Our analysis of the autophagy cluster genes increased the number of experimentally defined fission yeast autophagy factors from 14 to 23, and the identification of Atg10, Atg16, and Atg14 completed the roster of expected core autophagy components. We were, therefore, afforded an opportunity to comprehensively characterize the autophagy machinery in this organism for the first time. To survey the properties of fission yeast autophagy factors, we expressed them as fluorescent protein-tagged forms under the control of their endogenous promoters, and examined their subcellular localization by live cell imaging.

**Figure 3:**
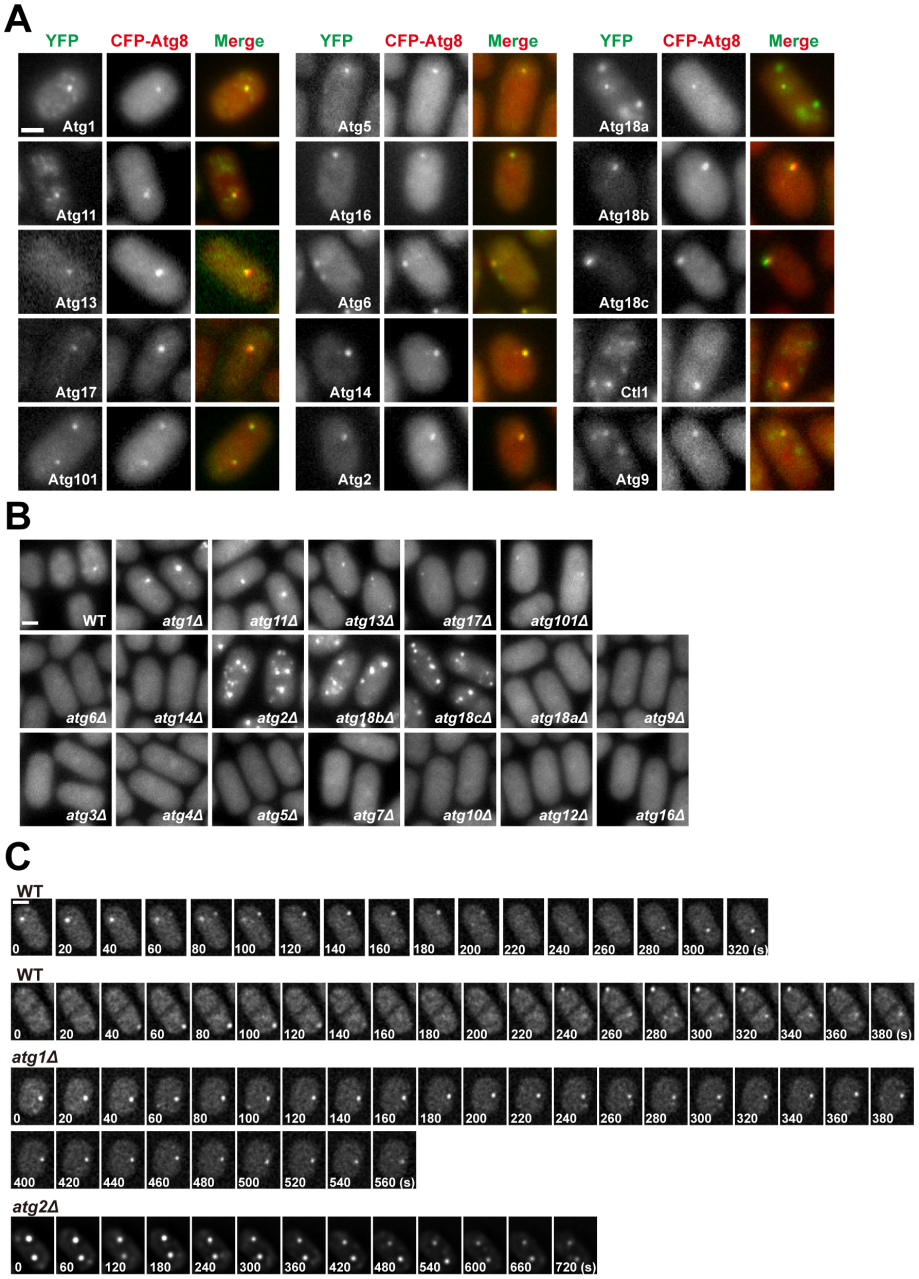
Subcellular localization of fission yeast autophagy factors. (A) Fifteen Atg proteins colocalized with CFP-Atg8 at cytoplasmic puncta induced by starvation. Images were acquired 2 h after starvation. (B) The distribution of CFP-Atg8 in *atg* mutants. Images were acquired 3 h after starvation. (C) Time-lapse analysis of CFP-Atg8 puncta in wild type, *atg1*Δ, and *atg2*Δ cells. Bars, 3 µm.

Atg8 is the only fission yeast Atg protein whose localization has been investigated [3], [13]. As reported by previous studies, we found that nitrogen starvation triggered the formation of bright CFP-Atg8 puncta in the cytoplasm. Co-expressing other autophagy proteins tagged with YFP in the CFP-Atg8 strain showed that 14 Atg proteins and Ctl1 colocalized with Atg8 on the punctate structure (Figure 3A). In *S. cerevisiae*, the same set of Atg proteins also colocalize at a punctuate structure, which has been termed the pre-autophagosomal structure or phagophore assembly site (PAS) [29]–[31]. Because of the similarity in the way Atg proteins assemble together, we propose that the structure where fission yeast Atg proteins colocalize during starvation is the counterpart of PAS in budding yeast, and will refer to it as PAS hereafter.

The majority of the PAS-localizing fission yeast Atg proteins do not accumulate on distinct subcellular structures under non-starvation conditions. The exceptions are Atg1, Atg11, Atg6, Atg18a, Atg9, and Ctl1. In vegetatively growing cells, Atg1, Atg11 and Atg18a were observed on the vacuole membrane (Figure S5A). Atg18a also formed puncta co-localizing with an endosomal marker (Figure S5B). Atg6 was observed on punctate structures labeled by an endosomal marker as well, presumably reflecting its role in the vacuolar protein sorting pathway [28] (Figure S5C). The localization patterns of Atg9 and Ctl1 will be described below.

In *S. cerevisiae*, the localization of Atg8 at PAS is influenced by many other autophagy factors [29], [31]. To assess how fission yeast autophagy factors act, we analyzed the localization of CFP-Atg8 in *atg* mutants during starvation (Figure 3B). Mutants of the Atg8 conjugation system, *atg3*Δ, *atg4*Δ, *atg5*Δ, *atg7*Δ, *atg10*Δ, *atg12*Δ, and *atg16*Δ, completely abolished Atg8 puncta formation, so did the PI3K mutants *atg6*Δ and *atg14*Δ. In contrast, Atg8 puncta were readily detected in *atg1*Δ, *atg11*Δ, *atg13*Δ, *atg17*Δ, *atg101*Δ, *atg2*Δ, *atg18b*Δ, and *atg18c*Δ. These eight mutants can be classified into three groups based on the number, intensity, and emergence timing of the Atg8 puncta. Group 1 consists of *atg1*Δ and *atg11*Δ, in which Atg8 puncta appeared relatively normal in the first hour after starvation, but their numbers did not decline afterwards as happened in the wild type. Group 2 consists of *atg13*Δ, *atg17*Δ, and *atg101*Δ, which lacked obvious Atg8 puncta during the first hour after starvation, and the puncta emerged later appeared dimmer than those found in the wild type. Group 3 consists of *atg2*Δ, *atg18b*Δ, and *atg18c*Δ, in which the Atg8 puncta were much more numerous than in the wild type at all time points, and some of the puncta were notably brighter than those in the wild type.

Despite the superficial resemblance of the snapshot images of the Atg8 puncta in *atg1*Δ cells and wild type cells, time-lapse imaging analysis showed that unlike wild type cells, in which Atg8 puncta were dynamic structures with durations mostly in the range of 100 to 200 seconds, Atg8 puncta persisted much longer in *atg1*Δ cells (Figure 3C). This is similar to the observations in *S. cerevisiae* [32], [33]. In addition, we found that Atg8 puncta in *atg2*Δ cells were also long-lasting structures (Figure 3C).

### Atg18a is required for the recruitment of the Atg12–Atg5·Atg16 complex to PAS

**Figure 4:**
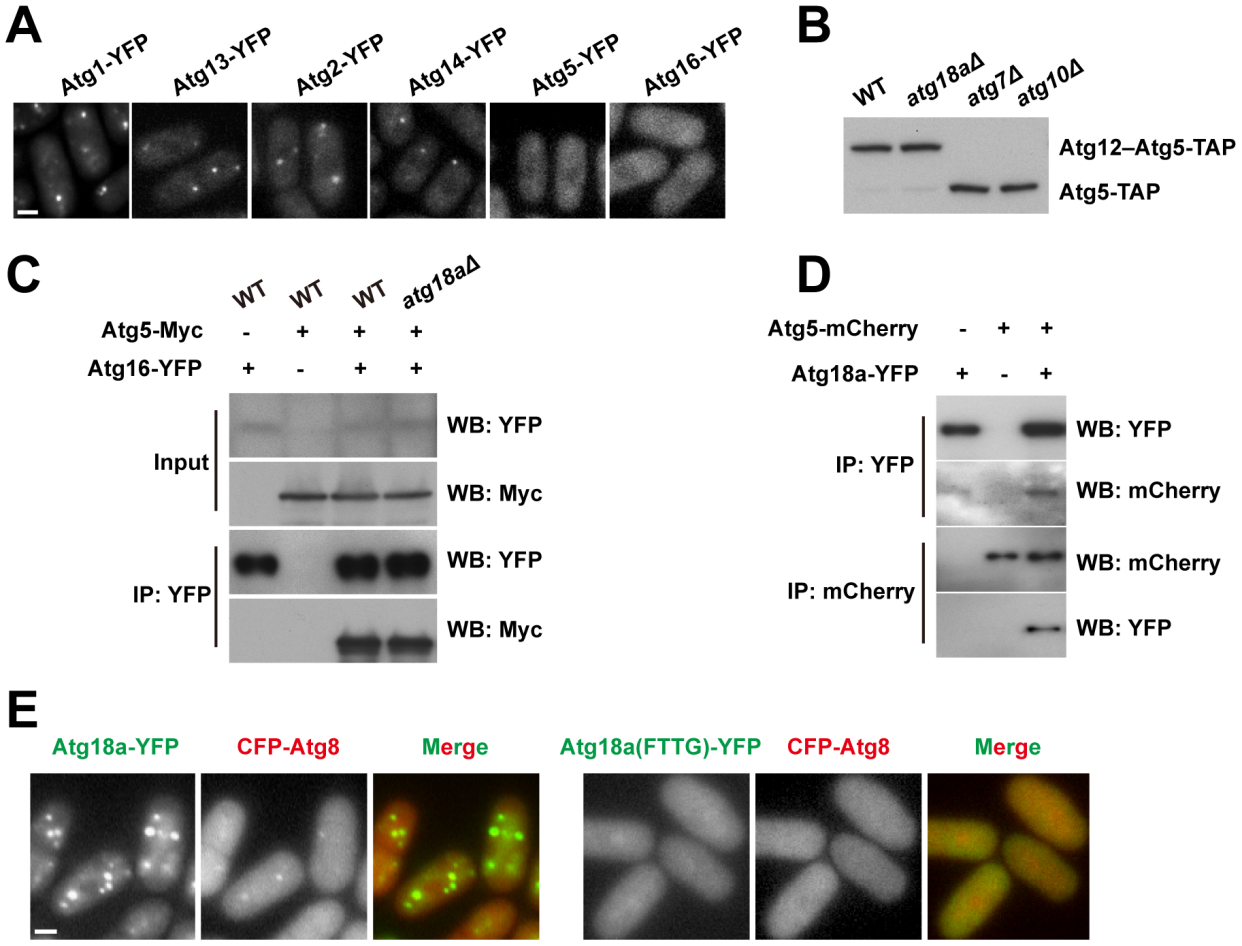
Atg18a is required for the PAS targeting of the Atg12–Atg5·Atg16 complex. (A) *atg18a*Δ abolished the starvation-induced puncta formation by Atg5 and Atg16. (B) Atg12–Atg5 conjugation is normal in *atg18a*Δ cells. (C) The interaction between Atg5 and Atg16 is intact in *atg18a*Δ cells. Input, 1%; IP, 20%. (D) Atg5 and Atg18a co-immunoprecipitated with each other. (E) Mutating the FRRG motif in Atg18a abolished its own puncta and the Atg8 puncta in starved cells. Bars, 3 µm.

One particularly intriguing observation was the lack of Atg8 puncta in *atg18a*Δ cells (Figure 3B), suggesting that Atg18a plays a role different from that of the other two Atg18/WIPI paralogs, Atg18b and Atg18c. To assess how *atg18a*Δ may affect the PAS organization, we examined the localization of several representative Atg proteins in this mutant (Figure 4A). Atg1, Atg13, Atg14, and Atg2 still formed puncta in starved *atg18a*Δ cells. In contrast, neither Atg5 nor Atg16 formed detectable puncta. Thus, *atg18a*Δ blocked the recruitment of Atg5 and Atg16 to PAS, and probably as a consequence, indirectly abolished the PAS localization of Atg8.

We have shown that in *S. pombe*, Atg5 mainly exists in the form of Atg12–Atg5 conjugate, and physically interacts with Atg16 (Figure 2C). Neither Atg12–Atg5 conjugate formation (Figure 4B), nor the interaction between Atg5 and Atg16 (Figure 4C), was affected by *atg18a*Δ. Thus, the Atg12–Atg5·Atg16 complex remains intact in *atg18a*Δ cells, and the localization defect is probably due to a failure to recruit this complex as a whole to PAS.

We hypothesized that Atg18a may physically interact with the Atg12–Atg5·Atg16 complex. To test this idea, we performed co-IP experiments and found that, indeed, Atg5 was co-precipitated with Atg18a, and in a reciprocal IP, Atg18a was co-precipitated with Atg5 (Figure 4D). Thus, Atg18a may serve as a binding platform for the recruitment of the Atg12–Atg5·Atg16 complex to PAS.

The Atg18 family proteins bind PI3P in a manner dependent on a conserved FRRG motif [34], [35]. When the FRRG motif in Atg18a was mutated to FTTG, the protein became diffusely distributed and could no longer support the puncta formation by Atg8 (Figure 4E). Together, our data support a sequential recruitment model in which Atg18a is targeted to PAS by PI3P binding, and then in turn recruits the Atg12–Atg5·Atg16 complex.

### Ctl1 is required for starvation-induced autophagy

**Figure 5:**
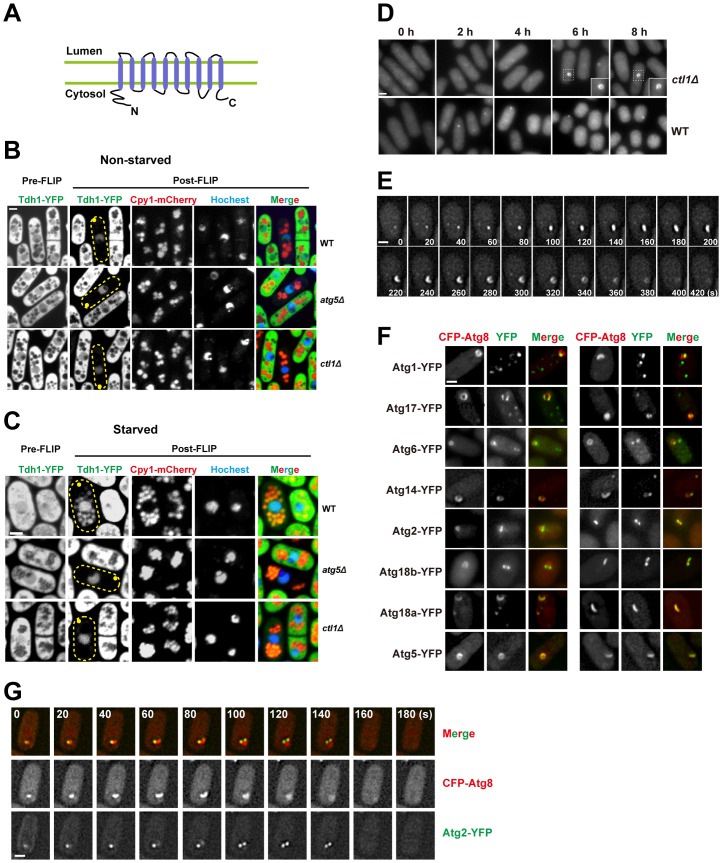
Ctl1 is required for autophagic transport and normal PAS organization. (A) The predicted membrane topology of Ctl1. (B,C) A fluorescence loss in photobleaching (FLIP) assay revealed the immobilized and non-diffusible pools of Tdh1-YFP. Yellow dots mark the sites of photobleaching. (B) In non-starved cells, only nuclear YFP signal remained in post-FLIP images. (C) In starved cells, vacuolar YFP signal was observed in post-FLIP images of wild-type, but not *atg5*Δ or *ctl1*Δ cells. (D) Ring-shaped and C-shaped structures labeled by CFP-Atg8 appeared in *ctl1*Δ cells after prolonged starvation. (E) Time-lapse images of a *ctl1*Δ cell. (F) The localization patterns of other Atg proteins in *ctl1*Δ cells containing CFP-Atg8-labeled structures. (G) Time-lapse images of a *ctl1*Δ cell expressing both CFP-Atg8 and YFP-Atg2. Bars, 3 µm.

We gave the previously uncharacterized gene *SPCC1682.11c* the name *ctl1* because it encodes the sole member of the choline transporter-like (CTL) protein family (Pfam PF04515) in *S. pombe*. This protein family is ubiquitous in eukaryotes, with one member in *S. cerevisiae* (Pns1), one member in *C. elegans* (CHTL-1), two members in *D. melanogaster*, and five members in humans [36] (Figure S6). One vertebrate CTL protein, CTL1/SLC44A1, was shown to be a choline transporter on the plasma membrane and in mitochondria [37], [38]. However, *S. cerevisiae* Pns1 and *C. elegans* CHTL-1 do not act as choline transporters [39], [40]. Thus, choline transport does not appear to be a universal function of CTL proteins. Like other members of the CTL family, *S. pombe* Ctl1 is predicted to be a multi-transmembrane protein, with several methods agreeing on the same prediction that Ctl1 contains 10 transmembrane helices with both its N terminus and C terminus facing the cytoplasm (Figure 5A).

To corroborate the result of our CFP-Atg8 processing assay, we used a fluorescence loss in photobleaching (FLIP) assay to monitor the non-specific autophagy of an abundant cytosolic protein Tdh1, which is the major form of glyceraldehyde-3-phosphate dehydrogenase (GAPDH) in fission yeast [41]. In this assay, the fluorescence signal of the diffusible pool of Tdh1-YFP is depleted by repetitive photobleaching of a small region near one tip of the cell. If Tdh1-YFP is trapped or immobilized in certain cellular compartments and cannot freely diffuse to the site of photobleaching, such cellular compartments should stand out in post-FLIP images due to the remaining YFP signal. In non-starved cells, the only compartment with visible YFP signal in post-FLIP images is the nucleus (Figure 5B), perhaps due to the reported association of Tdh1 with RNA polymerase II [42]. Upon starvation, in post-FLIP images of wild-type cells (Figure 5C), YFP signal became detectable in vacuoles, which were labeled by mCherry-tagged Cpy1 (carboxypeptidase Y, or CPY), a vacuolar lumenal protein [43]. The starvation-induced vacuolar YFP signal is due to the autophagic delivery of Tdh1-YFP, because deletion of *atg5* abolished such signal (Figure 5C). *ctl1*Δ also blocked the starvation-induced vacuolar targeting of Tdh1-YFP (Figure 5C). This result confirmed that Ctl1 is required for non-specific autophagy.

### Ctl1 is required for the normal organization of PAS

To understand how Ctl1 contributes to autophagy, we examined the localization of Atg8 in *ctl1*Δ cells. In wild type cells, the level of starvation-induced CFP-Atg8 puncta peaked at around 1 h after starvation. However, in *ctl1*Δ cells, no Atg8 puncta were observed in the first few hours after starvation (Figure 5D). After prolonged starvation (>4 h), Atg8 became concentrated on cytoplasmic structures in *ctl1*Δ cells. Remarkably, some of these CFP-Atg8 labeled structures are not dot-like as seen in wild type, but rather C-shaped or ring-shaped (Figure 5D). Time-lapse analysis showed that these distinctly shaped structures represent different stages of a dynamic process, in which a CFP-Atg8 labeled structure first emerges as a dot, then elongates and bends into a C-shape, and subsequently grows to form a ring before its eventual disappearance, in a total time frame of several minutes (Figure 5E). The C shape may correspond to a cup-like structure in three-dimensional space, and the ring shape may correspond to a hollow sphere-like structure.

Interestingly, YFP-tagged Atg proteins localized to different regions of the Atg8-labeled structure in *ctl1*Δ cells: Atg5 perfectly co-localized with Atg8, whereas Atg1, Atg17, Atg6, Atg14, Atg2, and Atg18b localized to sub-regions of the Atg8-labeled structures, with Atg2 and Atg18b concentrating at the tips of the expanding structures (Figure 5F and G). Thus, even though this structure is a pathological outcome of the loss of Ctl1, it resembles the PAS in that many Atg proteins localize on it. We suspect that the distinct localization patterns of the Atg proteins on this structure may reflect their intrinsic properties. Consistent with this idea, we found that unlike Atg2 and Atg18b, Atg18a perfectly colocalized with Atg8 on this structure (Figure 5F), echoing its role in promoting the PAS localization of Atg5 and Atg8.

### Ctl1 and Atg9 interact with each other and influence each other's localization

**Figure 6:**
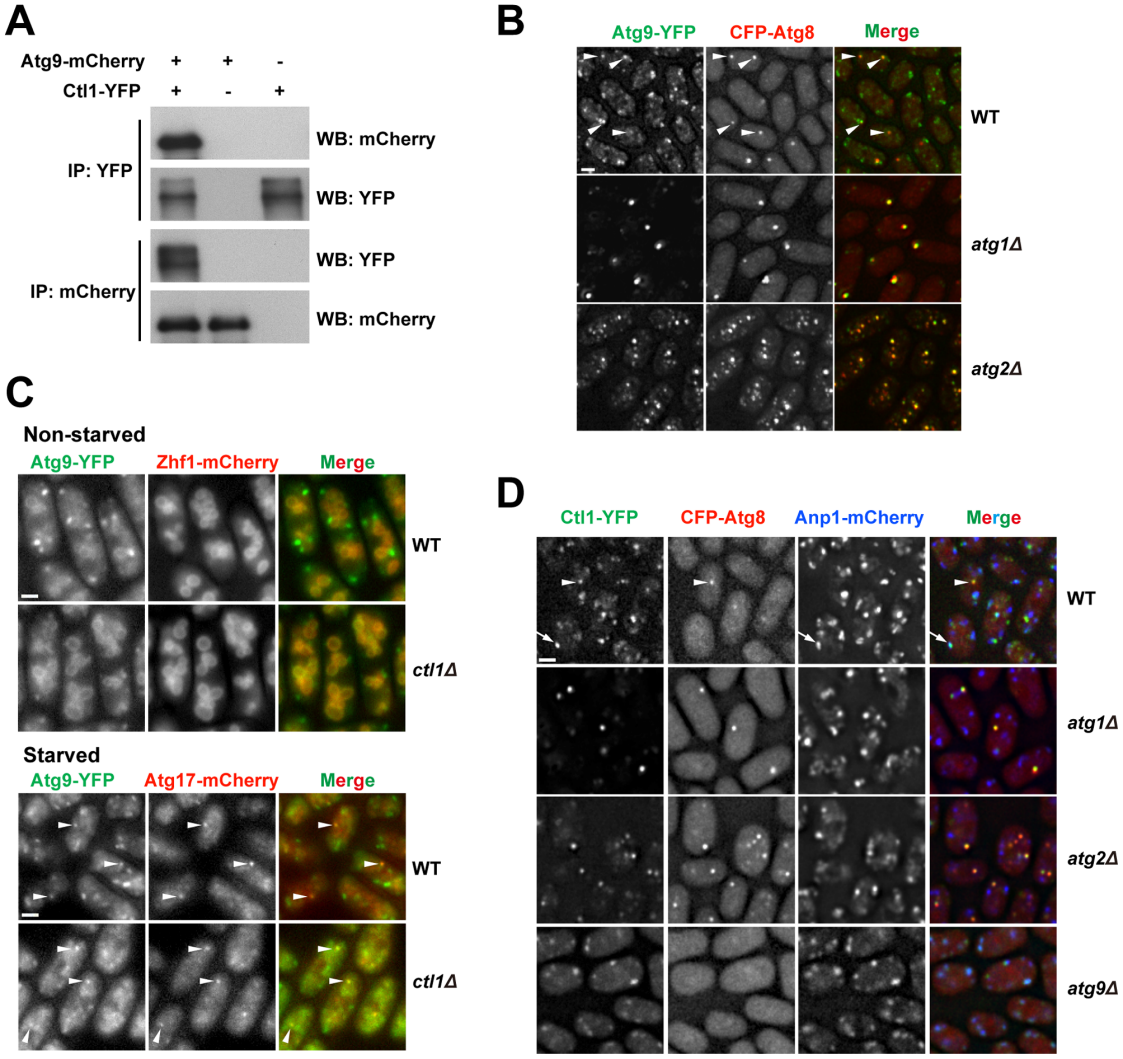
Ctl1 and Atg9 interact with each other and influence each other's localization. (A) Atg9 and Ctl1 co-immunoprecipitated with each other. (B) Localization patterns of Atg9 and Atg8 in starved cells. Arrowheads point to the puncta where Atg9 and Atg8 colocalized in the wild-type cells. (C) *ctl1*Δ altered the localization pattern of Atg9 in non-starved cells. Zhf1 is a vacuole membrane marker [77], and Atg17 is a PAS marker. Arrowheads point to puncta where Atg9 and Atg17 colocalized. (D) Localization patterns of Ctl1 in starved cells. Atg8 and Anp1 are PAS and Golgi markers, respectively. The arrowhead points to a punctum where Ctl1 and Atg8 colocalized. The arrow points to a punctum where Ctl1 and Anp1 colocalized. Bars, 3 µm.

To determine whether Ctl1 is associated with other autophagy factors, we performed affinity purification coupled with mass spectrometry analysis and found that Atg9 co-purified with Ctl1 (unpublished data). Reciprocal co-IP experiments demonstrated that Ctl1 and Atg9 indeed interact with each other (Figure 6A).

In *S. cerevisiae*, Atg9 cycles between PAS and non-PAS compartments, and the retrograde trafficking of Atg9 from PAS requires Atg1 and Atg2 [44]–[46]. Similarly, we found that in *S. pombe*, Atg9-YFP localized to punctate cytoplasmic structures, and partially co-localized with Atg8 puncta during starvation (Figure 6B). In starved *atg1*Δ or *atg2*Δ cells, Atg9 puncta almost completely overlapped with Atg8 puncta (Figure 6B), suggesting that recycling of Atg9 from PAS requires Atg1 and Atg2, like in *S. cerevisiae*.

The Ctl1-Atg9 interaction prompted us to examine whether Ctl1 influences the distribution of Atg9. In non-starved wild-type cells, besides the punctate structures, we also found weak Atg9-YFP signal on the vacuole membrane (Figure 6C). In non-starved *ctl1*Δ cells, Atg9 puncta no longer stood out, whereas the vacuole membrane signal was more noticeable, suggesting that Atg9 may be partially mislocalized (Figure 6C). In starved *ctl1*Δ cells, Atg9 puncta became more prominent, and some of them co-localized with mCherry-tagged Atg17 (Figure 6C), suggesting that Atg9 can still traffic to the PAS in the absence of Ctl1.

Similar to Atg9, Ctl1 tagged with YFP localized to cytoplasmic punctate structures, and upon starvation, a fraction of Ctl1-YFP puncta colocalized with CFP-Atg8 puncta (Figure 6D). In addition, Ctl1 became largely restricted to PAS in starved *atg1*Δ or *atg2*Δ cells (Figure 6D), suggesting that like Atg9, Ctl1 is recycled from PAS in an Atg1 and Atg2-dependent manner.

In *S. cerevisiae*, it has been shown that Atg9 traffics from Golgi apparatus to PAS via small vesicular carriers [46]. As Ctl1 and Atg9 may share similar cycling routes, we monitored the spatial relationship between Ctl1 and a Golgi marker, Anp1 [47]. In starved wild-type cells, Ctl1 puncta partially colocalized with Anp1, whereas in starved *atg9*Δ cells, nearly 100% of Ctl1 puncta colocalized with Anp1 (Figure 6D), indicating that Ctl1 travels from Golgi to PAS in an Atg9-dependent manner.

### Fsc1 localizes to the vacuole membrane

**Figure 7:**
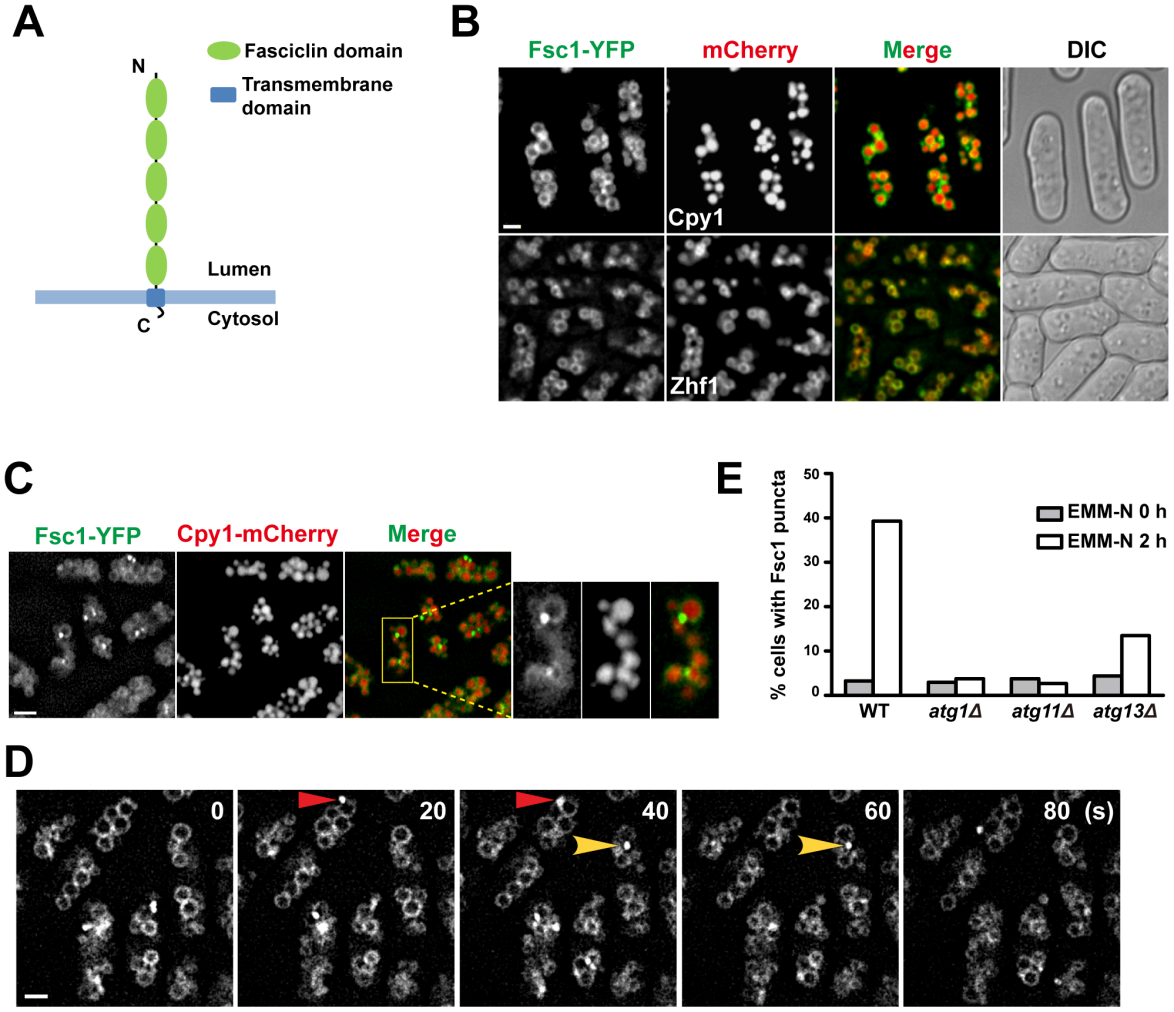
Fsc1 localizes to the vacuole membrane and forms starvation-induced puncta. (A) The predicted membrane topology and domain organization of Fsc1. (B) Localization of Fsc1 in non-starved cells. Cpy1 and Zhf1 are vacuole lumen and vacuole membrane markers, respectively. Bar, 3 µm. (C) Localization of Fsc1 in starved cells. Bar, 6 µm. (D) Time-lapse images of Fsc1 puncta induced by starvation. Bar, 3 µm. (E) Fsc1 puncta induced by starvation are dependent on Atg1, Atg11, and partially dependent on Atg13. Two hundred cells were examined for each data point.

We gave the previously uncharacterized gene *SPAC22H12.05c* the name *fsc1* because it encodes a protein containing five fasciclin domains (Pfam PF02469) (Figure 7A and Figure S7). Fasciclin domain-containing proteins exist in animals, fungi, plants, bacteria, and cyanobacteria [48]. In animals and plants, this type of protein is usually found at the cell surface and mediate cell adhesion [49], [50]. However, one mammalian fasciclin domain protein, stabilin-1, was reported to have intracellular trafficking roles [51].

Fsc1 is predicted to be a type I transmembrane protein, with the bulk of its amino acids exposed in the lumenal/extracellular space (Figure 7A). In vegetatively growing cells, Fsc1 tagged at its C-terminus with YFP localized to the vacuole membrane (Figure 7B). Interestingly, upon starvation, bright puncta of Fsc1-YFP were observed on the vacuolar rim of a small number of vacuoles (Figure 7C), indicating a dramatic concentration of Fsc1 at special sites on the vacuole membrane. The starvation-induced Fsc1 puncta were dynamic structures with durations of less than a minute (Figure 7D), and they were abolished in *atg1*Δ and *atg11*Δ cells, and diminished in *atg13*Δ cells (Figure 7E). No overlap between Fsc1 puncta and Atg8 puncta was observed (Figure S8), indicating that these two types of starvation-induced structures are spatially distinct entities.

### Fsc1 is required for the fusion of autophagosomes with vacuoles

**Figure 8:**
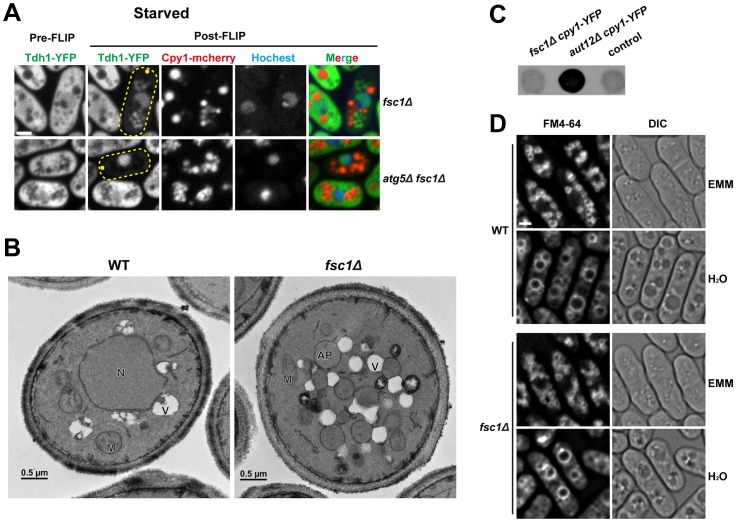
Fsc1 is required for autophagosome-vacuole fusion. (A) In starved *fsc1*Δ cells, Tdh1-YFP entered closed cytoplasmic membrane structures, which are not vacuoles. These structures are dependent on Atg5, thus are likely autophagosomes. Bar, 3 µm. (B) TEM analysis of starved wild-type and *fsc1*Δ cells. N, nucleus; M, mitochondrion; V, vacuole; AP, autophagosome. (C) Unlike the mutant lacking a general vacuolar fusion factor Aut12, *fsc1*Δ cells did not secret Cpy1-YFP, which was detected by a colony blot assay with an antibody recognizing YFP. The control is a strain not expressing Cpy1-YFP. (D) *fsc1*Δ did not affect homotypic vacuole fusion occurring when cells were shifted from EMM medium to water. Vacuoles were stained with the vital dye FM4-64. Bar, 3 µm.

The fact that Fsc1 localizes to the destination compartment of autophagic trafficking led us to hypothesize that Fsc1 may be required for a late step of autophagy, the fusion between autophagosomes and vacuoles. To test this idea, we applied the Tdh1-YFP FLIP assay to *fsc1*Δ cells. Upon starvation, YFP signal in round cytoplasmic structures became visible in post-FLIP images of *fsc1*Δ cells (Figure 8A). However, unlike wild-type cells (Figure 5C), the YFP signal in *fsc1*Δ cells did not overlap with Cpy1-mCherry. Thus, Tdh1 entered a closed membrane compartment but did not reach the vacuole. Such YFP-labeled structures were absent in the post-FLIP images of *fsc1Δ atg5*Δ cells, suggesting that the Tdh1-containing membrane structures accumulated in *fsc1*Δ cells are autophagosomes.

To verify the results obtained by the FLIP assay, we performed transmission electron microscopy (TEM) analysis. In starved *fsc1*Δ cells, we observed the accumulation of spherical membrane structures whose lumenal contents had the same electron opacity as the cytosol, suggesting that they are autophagosomes (Figure 8B). Moreover, TEM images showed that autophagosomes in these cells were often in extensive contact with vacuoles, suggesting that docking of autophagosomes onto vacuoles had occurred but membrane fusion was blocked.

To our knowledge, all known autophagosome-vacuole fusion factors in *S. cerevisiae*, such as Mon1/Aut12 and Vam3, are required not only for autophagosome-vacuole fusion, but also for other vacuolar fusion events, such as those occurring in the CPY trafficking [52], [53], and homotypic vacuole fusion [54]. In contrast, Fsc1 appears to be dispensable for these processes, as unlike *aut12*Δ cells, Cpy1 was not missorted to cell surface in *fsc1*Δ cells (Figure 8C), and homotypic vacuole fusion induced by hypo-osmotic stress occurred normally in *fsc1*Δ cells (Figure 8D) [55]. Thus, Fsc1 is specifically required for autophagosome-vacuole fusion.

## Discussion

In this study, we quantitatively assessed the mating efficiencies of the fission yeast deletion library strains under 19 different mating conditions, and generated an extensive phenotyping dataset that allows hundreds of genes to be clustered in a way that reflects their functional relationships. The autophagy gene cluster represents a comprehensive inventory of fission yeast autophagy factors. Through systematic analyses of these autophagy factors, we uncovered novel autophagy mechanisms, gained insights into how autophagy pathway has evolved, and established the fission yeast as a model for deciphering the inner workings of the autophagy machinery.

### Organization of PAS in fission yeast versus budding yeast

In *S. cerevisiae*, PAS is the site where the Atg proteins involved in autophagosome formation assemble together, as observed by fluorescence microscopy. Here, we identified a similar entity in *S. pombe* by live cell imaging of fluorescent protein-tagged Atg proteins, and named it PAS due to its resemblance to the PAS in *S. cerevisiae*. The similarities include: (1) In both species, PAS is a dot-like structure whose finer details cannot be resolved by conventional light microscopy; (2) At any given time during starvation, in the majority of PAS-containing cells, only one PAS punctum can be observed; (3) The same set of Atg proteins can be colocalized at PAS, with the exception of Atg29 and Atg31, which are absent in *S. pombe*, and Atg101, which is absent in *S. cerevisiae*; (4) PAS is a dynamic structure with a duration in the range of minutes, as revealed by the time-lapse analysis of Atg8 puncta; (5) The assembly of Atg proteins at PAS is controlled in a hierarchical manner, with Atg8 being one of the most downstream factors, whose recruitment to PAS or dynamics at PAS is altered in the mutants defective in any other PAS-localizing Atg proteins.

There are also notable differences between these two organisms in terms of PAS organization and the roles of PAS-localizing Atg proteins: (1) In *S. cerevisiae*, a constitutive biosynthetic route termed cytoplasm-to-vacuole targeting (Cvt) pathway utilizes the Atg proteins to transport cytosolic hydrolases into the vacuole [56], and thus the assembly of Atg proteins at PAS occurs under nutrient-rich conditions; in contrast, PAS cannot be detected in *S. pombe* under nutrient-rich conditions, presumably due to the lack of the Cvt pathway, whose key factors Ape1 and Atg19 do not have apparent homologs in *S. pombe*; (2) Atg11 in *S. cerevisiae* is dispensable for starvation-induced autophagy, whereas Atg11 in *S. pombe* is essential for starvation-induced autophagy and appears to have a closer relationship with Atg1 than the other putative Atg1 regulators, consistent with a proposition that *S. pombe* Atg11 may be more similar to mammalian FIP200 than to budding yeast Atg11 [12]; (3) There are three Atg18/WIPI proteins in each species, but only one of the three paralogs in *S. cerevisiae* (Atg18/Svp1) is essential for starvation-induced autophagy, whereas all three paralogs in *S. pombe* are needed for starvation-induced autophagy.

### Functions of the Atg18/WIPI proteins

We found that the mutants of fission yeast Atg18 paralogs exhibited different phenotypes, with *atg18a*Δ abolishing the Atg8 puncta, and *atg18b*Δ or *atg18c*Δ elevating the levels of Atg8 puncta. This is analogous to the situation in mammalian cells, where LC3 (a mammalian homolog of Atg8) puncta increased upon the depletion of either WIPI1 or WIPI4 but decreased upon the depletion of WIPI2 [57]–[59]. Such functional distinctions cannot be readily explained by the phylogenetic relationships among Atg18/WIPI proteins (Figure S9 and S10). In mammals, WIPI1 and WIPI2 are much more similar to each other than to WIPI4, and in *S. pombe*, Atg18b and Atg18c do not show significantly higher sequence homology to each other than to Atg18a.

Our analysis suggests that the lack of Atg8 puncta in *atg18a*Δ cells is due to a defect in the PAS targeting of the Atg12–Atg5·Atg16 complex, which physically interacts with Atg18a. To our knowledge, this is the first time a physical interaction between a WIPI/Atg18 protein and the Atg12–Atg5·Atg16 complex has been observed. Similar interactions may underlie the roles of *S. cerevisiae* Atg18 and its paralog Atg21 in promoting the PAS localization of Atg5 and Atg16 [35], [60], and the role of mammalian WIPI2 in the recruitment of LC3 to the omegasome, which may be the equivalent of PAS in mammalian cells [57].

As Atg18a accumulates on subcellular structures other than PAS, it probably cooperates with additional factors for the specific targeting of Atg12–Atg5·Atg16 to PAS. Atg2 is unlikely to be such a factor, as its mutant behaved like *atg18b*Δ and *atg18c*Δ.

### The role of Ctl1 in autophagy

Ctl1 is a novel autophagy factor uncovered by our screens. Our phylogenetic analysis showed that fungal CTL proteins fall into two clades, with *S. pombe* Ctl1 in one clade, and *S. cerevisiae* Pns1 in the other (Figure S6). Pns1, whose function is unknown, has been localized at the plasma membrane [61], and it does not appear to be required for starvation-induced autophagy (unpublished data). Thus, among the fungal CTL proteins, perhaps only the ones falling into the same clade as Ctl1 are autophagy factors. As fungal species in many lineages have both Ctl1-like and Pns1-like proteins (Figure S6), these two types of proteins might have co-existed in the common ancestor of fungi, but one of them was lost in the lineage leading to *S. cerevisiae*, while the other was lost in the lineage leading to *S. pombe*.

In *ctl1*Δ cells, autophagosome formation appears to be defective, as we did not observe any cytoplasmic signal of Tdh1-YFP in post-FLIP images. The late emerging Atg8-labeled structures in *ctl1*Δ cells may be aberrant isolation membranes that cannot mature into completely sealed autophagosomes. Ctl1 may regulate the distribution of Atg proteins on the expanding isolation membrane, and in its absence, Atg proteins occupy different regions of the isolation membrane, instead of concentrating at one subregion.

It is interesting to note that the distinct localization patterns of Atg proteins we observed in *ctl1*Δ cells bear remarkable resemblance to the three types of Atg protein distribution patterns observed in *S. cerevisiae* when Ape1 is overexpressed [62]. In both *ctl1Δ S. pombe* cells and Ape1-overexpressing *S. cerevisiae* cells, Atg8 and Atg5 are distributed all over a cup-shaped structure, whereas Atg2 and an Atg18 family protein (Atg18 in *S. cerevisiae* and Atg18b in *S. pombe*) concentrate at the edge of this structure. In Ape1-overexpressing *S. cerevisiae* cells, Atg17, Atg6, and Atg14 localize to a subregion of the cup-shaped structure, termed vacuole-isolation membrane contact site (VICS); in *ctl1*Δ cells, these three proteins also localize to subregions of the cup-shaped structure. Thus, the spatial separation of Atg proteins under these two circumstances probably reflects evolutionarily conserved functional distinctions among the Atg proteins.

### Fsc1 and autophagosome-vacuole fusion

In *S. cerevisiae*, all known mutants blocking autophagosome-vacuole fusion are also defective for vacuolar fusion in the CPY and ALP pathways, as well as vacuole–vacuole homotypic fusion [63]. Thus, it is unclear whether there are mechanisms specifically regulating autophagosome-vacuole fusion in budding yeast. Here, we showed that, in *S. pombe*, a vacuole membrane protein Fsc1 is uniquely required for autophagosome-vacuole fusion, thus revealing a specific control of autophagic traffic at the vacuolar fusion step, and providing a molecular entry point for dissecting the mechanism of such a control. Fsc1 formed puncta on the vacuole membrane during starvation. We speculate that these structures may be in some way connected to autophagosome-vacuole fusion. For example, they may correspond to fusion-ready zones on the vacuole membrane, or the actual fusion sites, or special post-fusion structures.

Many fungal species have at least one protein sharing the exact same domain organization as Fsc1. The *S. cerevisiae* homolog of Fsc1 is Ylr001c (Figure S7), which like Fsc1, also localizes to the vacuole membrane [61]. However, Ylr001c seems to be dispensable for starvation-induced autophagy (unpublished data), perhaps due to functional redundancy in *S. cerevisiae*, or differences in vacuole physiology between the two organisms. One obvious difference is that an *S. cerevisiae* cell has one or a few large vacuoles, whereas an *S. pombe* cell has about 80 small vacuoles [55]. Thus, there may be a need for more elaborate fusion target selection in *S. pombe* to avoid overwhelming the degradative capacities of some vacuoles while leaving other vacuoles idle. Mammalian cells, where a large number of lysosomes are present in each cell, may share this need. Several lines of recent evidence suggest that autophagosome-lysosome fusion in mammalian cells utilizes mechanisms distinct from other lysosomal fusion events [64]–[66]. We expect that further analysis of Fsc1 may provide mechanistic insights relevant to autophagosome-lysosome fusion in mammalian cells.

## Materials and Methods

### Fission yeast strains and media

The fission yeast strains used in this study are listed in Table S4. Genetic methods for strain construction and composition of media are as described [20]. To construct a strain expressing CFP-Atg8 under the control of the endogenous promoter, we amplified by overlap-extension PCR the *atg8* promoter and the N-terminal region of the *atg8* ORF using primers 5′-GATCTAGAGAAGCGCTTATTTGTTTAC-3′, 5′-CGagatctTTGAGAACGCATGAGAACTCTCAAACTTCTTGC-3′, 5′-CTCATGCGTTCTCAAagatctCGTTCTCAATTCAAGG-3′, and 5′-GCGTCGACACCAACTGTAAGGTCAGATGG-3′. The final PCR product contained a BglII site (lowercase letters in the primer sequences) inserted near the start codon. The PCR product was digested with XbaI and SalI, and inserted into an integrating vector pJK148 [67]. DNA encoding the CFP tag was inserted into the BglII site to obtain the pJK148-CFP-Atg8 plasmid. The plasmid was linearized with SpeI, which cuts in the middle of the N-terminal region of the *atg8* ORF, and transformed into fission yeast. Most of the deletion strains used in this study were constructed by PCR amplifying the deletion cassettes in the Bioneer deletion strains and transforming the PCR product into strains from our lab strain collection. The exceptions are *atg1*, *atg3*, *atg6*, and *atg12*, whose deletion strains were made without the aid of Bioneer strains, by standard PCR-based gene targeting [68]. Strains expressing Atg proteins fused with the YFP-FLAG-His_6_ (YFH) tag under native promoters were constructed by an overlap-extension PCR approach [69], using the ORFeome plasmids as templates [70]. Strains expressing proteins fused with other tags were made by PCR-based tagging [68]. Tdh1-YFH was expressed from an ORFeome plasmid under the control of the *nmt1* promoter.

### Mating phenotype screens

Deletion strain pools of Bioneer version 1.0 haploid library (catalog number M-1030H) and Bioneer version 1.0 upgrade package (catalog number M-1030H-U) were constructed as described [19]. Frozen aliquots of the two mutant pools were thawed at room temperature, mixed together, washed once with YES medium, and pre-grown in YES or EMM medium for 3 generations at 30°C. An equal amount of log-phase wild-type *h^−^* strain (DY3984) grown in YES medium was mixed with the deletion mutants, and washed twice with water. The cell suspension was diluted to 100 OD_600_ units/ml in water, spotted on the solid mating medium, and incubated for 4 days. The mating mixtures were treated with 0.5% (v/v) glusulase overnight at room temperature, and the spores were purified with a Percoll step gradient [71]. The spore preparations were more than 99% pure as judged by microscopy. Genomic DNA extraction, barcode PCR, and Illumina sequencing were performed as described [19]. The sequencing data are publicly available at NCBI Sequence Read Archive (http://www.ncbi.nlm.nih.gov/sra/) under the accession number SRA068523. The data are split into 26 runs, which correspond to 4 input samples and 22 spore samples. Descriptions of the 26 runs are in Table S5.

### Barcode sequencing data analysis

Mating phenotype screen data were processed as described [19], with a few modifications. For read count normalization, we used the upper-quartile normalization method [72]. To avoid noises associated with very small read counts, for a MD score to be computed for a gene, we required the read count of at least one of its barcodes to be no smaller than 1/40 of the upper-quartile read count, and also no smaller than 12, in either the input sample or the spore sample. For a gene with a single barcode decoded, its MD score is the normalized log2 fold change (input versus spore) of that barcode. For a gene with both uptag and dntag decoded, its MD score is a weighted average of the normalized log2 fold change of the two barcodes, where the weight for a barcode is the ratio of the sum of the read counts of that barcode in input and spore samples to the sum of the read counts of both barcodes. To select mating defective mutants, we calculated for each gene a robust Z-score, which is the deviation of its MD score from the median MD score expressed in the number of the normalized interquartile range (NIQR). Tail area-based FDR values were calculated from the robust Z-scores using the software fdrtool version 1.2.8 [73]. Genes with FDR values <0.1 in all three screens performed under standard conditions were deemed the screen hits. GO term enrichment analysis was conducted with AmiGO version 1.8 using GO database release 2013-02-02 [74]. Hierarchical clustering analysis was performed using the correlation (uncentered) similarity metric and the complete linkage clustering method.

### CFP-Atg8 processing assay

Cell lysates were prepared using a post-alkaline extraction method [75]. Samples were separated by 12% SDS-PAGE and immunoblotted with an anti-GFP antibody (Roche).

### Atg12–Atg5 conjugate analysis and co-immunoprecipitation

The Peroxidase Anti-Peroxidase (PAP) soluble complex (Sigma) was used in immunoblotting to recognize the TAP tag fused to Atg5. For immunoprecipitation, cell lysates were made by glass bead beating. GFP-trap and RFP-trap agarose beads (ChromoTek) were used for immunoprecipitating YFP- and mCherry-tagged proteins, respectively.

### Light microscopy

Except for the FLIP assay, light microscopy was performed using a DeltaVision PersonalDV system (Applied Precision) equipped with a CFP/YFP/mCherry filter set (Chroma 89006 set) and a Photometrics CoolSNAP HQ2 camera. Images were acquired with a 100×, 1.4-NA objective, and analyzed with the SoftWoRx software.

### FLIP assay

Photobleaching of the Tdh1-YFP signal and image acquisition were carried out with a PerkinElmer Ultraview VoX spinning disk system, using a 100× objective. Image analysis was performed with the Volocity software.

### Electron microscopy

Cells were prepared for electron microscopy by fixation with glutaraldehyde and KMnO_4_ [76]. Samples were dehydrated with graded ethanol series, and embedded in Spurr's resin. Thin sections were stained with uranyl acetate and Sato's lead, and visualized on a transmission electron microscope.

## Supporting Information

Figure S1 Comparison between the results of the three screens conducted under standard mating conditions. (A) Scatter plots depicting the pair-wise comparisons between the screens. Dashed lines represent the FDR<0.1 cutoff. The 206 genes satisfying the cutoff in all three screens are highlighted in red. (B) A Venn diagram depicting the overlaps between the three sets of genes satisfying the FDR cutoff in individual screens.
(PDF)

Figure S2 A detailed view of the heat map shown in Figure 1F.
(PDF)

Figure S3 Fission yeast SPAC227.04 protein shares homology with Atg10 proteins in other species. Genbank accession numbers are gi|18594496 (*Homo sapiens*), gi|161076388 (*Drosophila melanogaster*), gi|71984851 (*Caenorhabditis elegans*), gi|30680332 (*Arabidopsis thaliana*), gi|19113870 (*Schizosaccharomyces pombe*), and gi|6322986 (*Saccharomyces cerevisiae*). Red arrowhead points to the catalytic cysteine. Black arrowheads point to the two residues suggested to play critical roles in catalysis [78].
(PDF)

Figure S4 Fission yeast SPBC405.05 protein shares homology with Atg16 proteins in other species. (A) Multiple sequence alignment of the Atg5-binding domain in Atg16 proteins. Open arrowheads point to the two residues important for the interaction between Atg5 and Atg16 in *S. cerevisiae* [79]. (B) Multiple sequence alignment of the coiled-coil domain (CCD) in Atg16 proteins. Filled arrowheads point to the four residues important for autophagic activity in *S. cerevisiae* [80]. (C) The domain organization of *S. cerevisiae* Atg16 protein (ScAtg16) and *S. pombe* Atg16 protein (SpAtg16). The domain boundaries of ScAtg16 is according to structural analysis [80]. The position of Atg5-binding domain in SpAtg16 is according to the alignment in (A). The position of CCD in SpAtg16 is as predicted by Marcoil using a probability threshold of 50% [81]. Genbank accession numbers are gi|124256480 (*Homo sapiens*), gi|62955681 (*Danio rerio*), gi|260796567 (*Branchiostoma floridae*), gi|198422508 (*Ciona intestinalis*), gi|28572018 (*Drosophila melanogaster*), gi|134117369 (*Cryptococcus neoformans*), gi|169844388 (*Coprinopsis cinerea*), gi|169625684 (*Phaeosphaeria nodorum*), gi|67515617 (*Aspergillus nidulans*), gi|19113100 (*Schizosaccharomyces pombe*), and gi|2497167 (*Saccharomyces cerevisiae*).
(PDF)

Figure S5 The subcellular localization of Atg1, Atg11, Atg18a, and Atg6 under non-starvation conditions. Bars, 3 µm. (A) Atg1, Atg11, and Atg18a colocalized with a vacuole membrane marker Zhf1. (B) Atg18a colocalized with an endosomal marker Hse1. (C) Atg6 colocalized with an endosomal marker Vps32.
(PDF)

Figure S6 Phylogenetic relationship between CTL family proteins in fungi. The CTL family proteins in 28 fungi species were identified by exhaustive search using PSI-BLAST at MPI Bioinformatics Toolkit web server [82]. Multiple sequence alignment was generated using MAFFT [83]. Phylogenetic tree was created with FastTree [84] and visualized using FigTree (http://tree.bio.ed.ac.uk/). CTL proteins from three metazoan species (human, *C. elegans*, and *D. melanogaster*) were used as outgroup for rooting the tree. Among the 39 fungal proteins, the ones showing closer relationship with fission yeast Ctl1 protein (NP_587804.1) are colored red; the ones showing closer relationship with budding yeast Pns1 protein (NP_014804.3) are colored green. The 11 species with two CTL proteins are marked by bold font.
(PDF)

Figure S7 Fasciclin domains in *S. pombe* Fsc1 and *S. cerevisiae* Ylr001c. (A) The domain organizations of Fsc1 and Ylr001c. (B) The alignment of the individual fasciclin domains in Fsc1 and Ylr001c with two fasciclin domains whose 3D structures have been solved. The alignment was generated and edited with Jalview [85]. Secondary structural elements of the fourth fasciclin domain of Drosophila fasciclin I (PDB 1O70) and the fourth fasciclin domain of human transforming growth factor-beta-induced protein ig-h3 (PDB 1X3B) were visualized together with the sequence alignment using the ESPript web server (http://espript.ibcp.fr/) [86].
(PDF)

Figure S8 Time-lapse images of a cell expressing Fsc1-YFP and CFP-Atg8. Bar, 3 µm.
(PDF)

Figure S9 The sequence alignment of Atg18/WIPI proteins. The alignment was generated and edited with Jalview [85]. Secondary structural elements of *K. lactis* Hsv2 (PDB 4EXV) were visualized together with the sequence alignment using the ESPript web server (http://espript.ibcp.fr/) [86]. The red bar denotes the FRRG motif involved in PI3P binding. The green and blue bars denote two sets of residues that are important for the Atg18-Atg2 interaction in *S. cerevisiae*, locating at the BC loop of blade 2 [87], and the loop connecting blade 2 and blade 3 [88], respectively. Genbank accession numbers of these proteins are listed in Figure S10.
(PDF)

Figure S10 Phylogenetic relationship between Atg18/WIPI proteins. The sequence alignment in Figure S9 was used for phylogenetic tree construction. The phylogenetic tree was created with FastTree [84] and visualized using FigTree (http://tree.bio.ed.ac.uk/). Atg18 homologs from Arabidopsis were used as outgroup for rooting the tree.
(PDF)

Table S1 The genes whose deletion mutants are mating defective under standard mating conditions.
(XLS)

Table S2 The mating conditions of the 22 screens.
(PDF)

Table S3 The MD scores from the 22 screens.
(XLS)

Table S4 The strains used in this study.
(PDF)

Table S5 The barcode sequencing data deposited at SRA.
(PDF)
