# Supplementary figures and images for: Global Analysis of Fission Yeast Mating Genes Reveals New Autophagy Factors
Source: PLoS Genet. 2013 Aug 8;9(8):e1003715. doi: 10.1371/journal.pgen.1003715 (PMC3738441; doi:10.1371/journal.pgen.1003715)

**A**

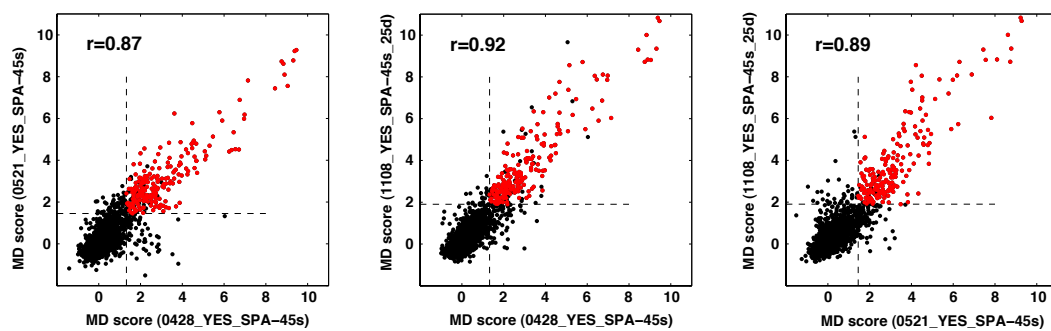

**B**

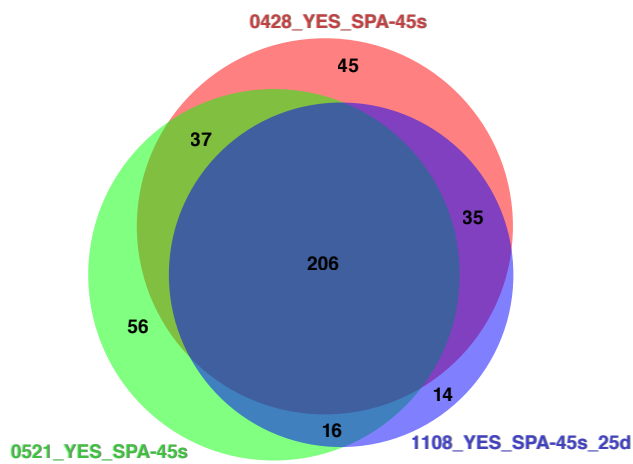

Supplement: Figure S1 — Comparison between the results of the three screens conducted under standard mating conditions. (A) Scatter plots depicting the pair-wise comparisons between the screens. Dashed lines represent the FDR<0.1 cutoff. The 206 genes satisfying the cutoff in all three screens are highlighted in red. (B) A Venn diagram depicting the overlaps between the three sets of genes satisfying the FDR cutoff in individual screens. (PDF) [file pgen.1003715.s001.pdf]

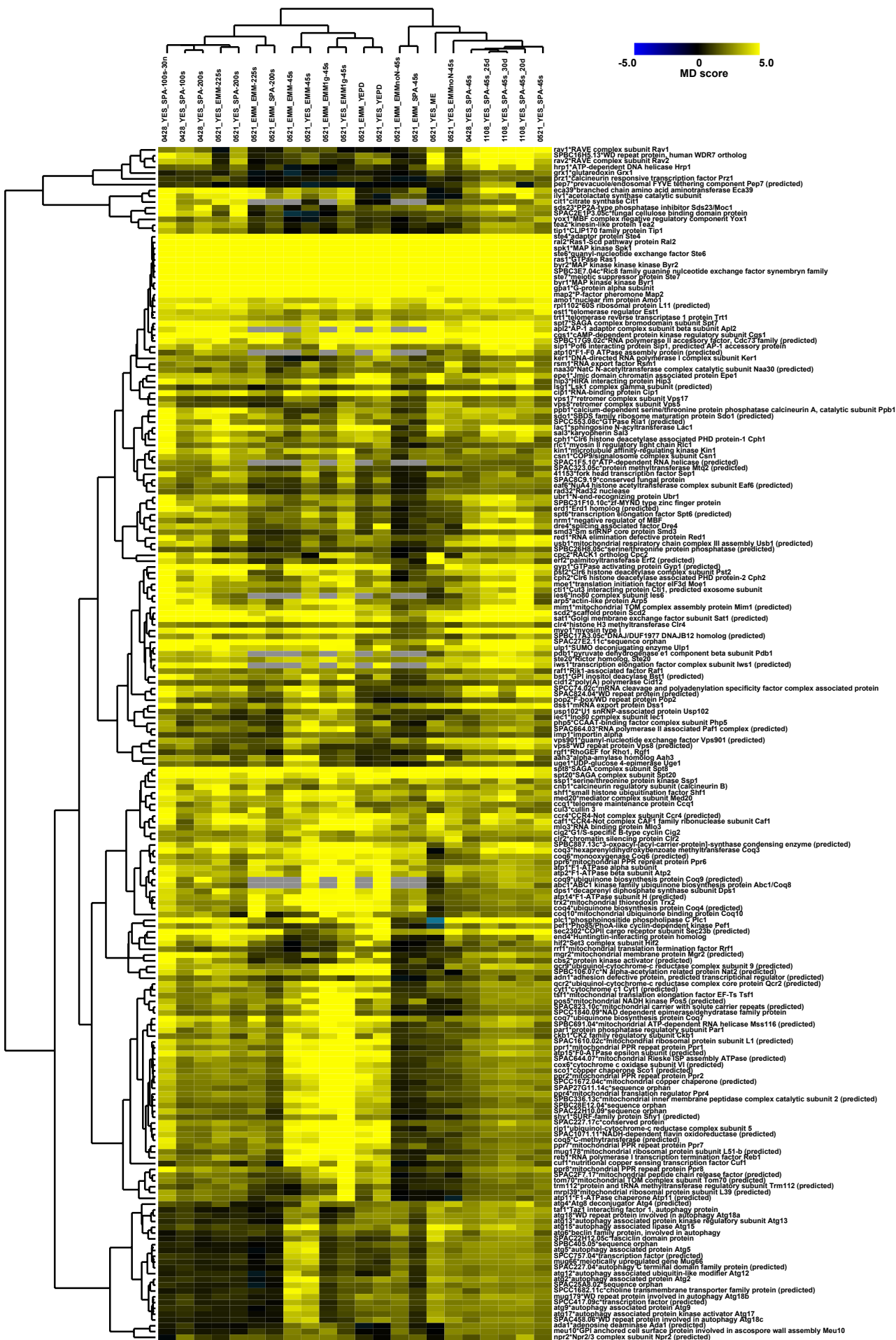

Supplement: Figure S2 — A detailed view of the heat map shown in Figure 1F. (PDF) [file pgen.1003715.s002.pdf]

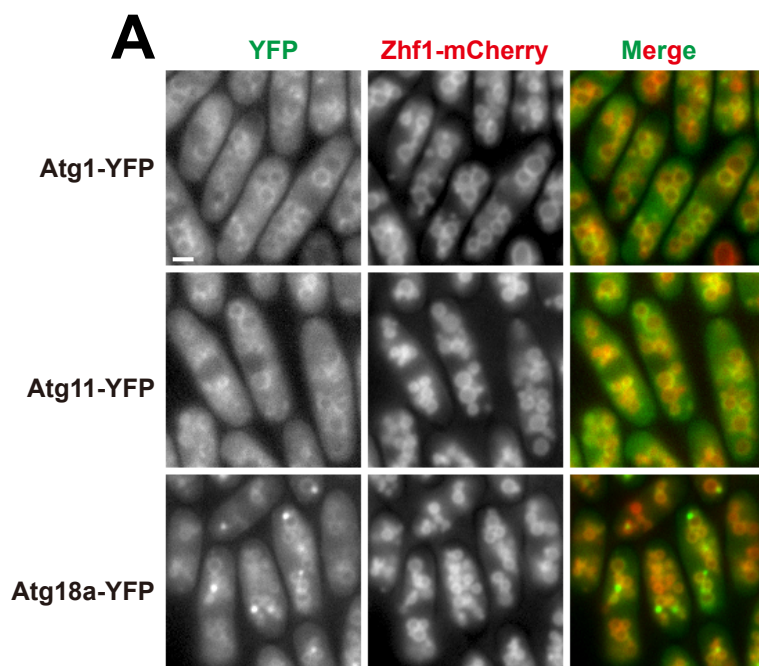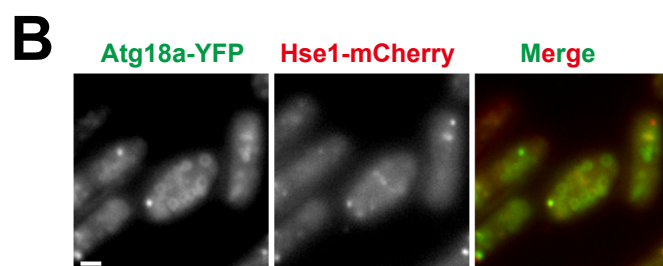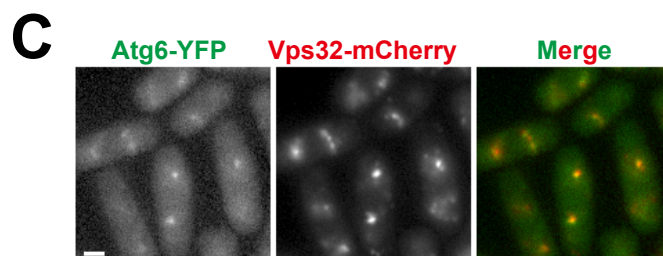

Supplement: Figure S5 — The subcellular localization of Atg1, Atg11, Atg18a, and Atg6 under non-starvation conditions. Bars, 3 µm. (A) Atg1, Atg11, and Atg18a colocalized with a vacuole membrane marker Zhf1. (B) Atg18a colocalized with an endosomal marker Hse1. (C) Atg6 colocalized with an endosomal marker Vps32. (PDF) [file pgen.1003715.s005.pdf]

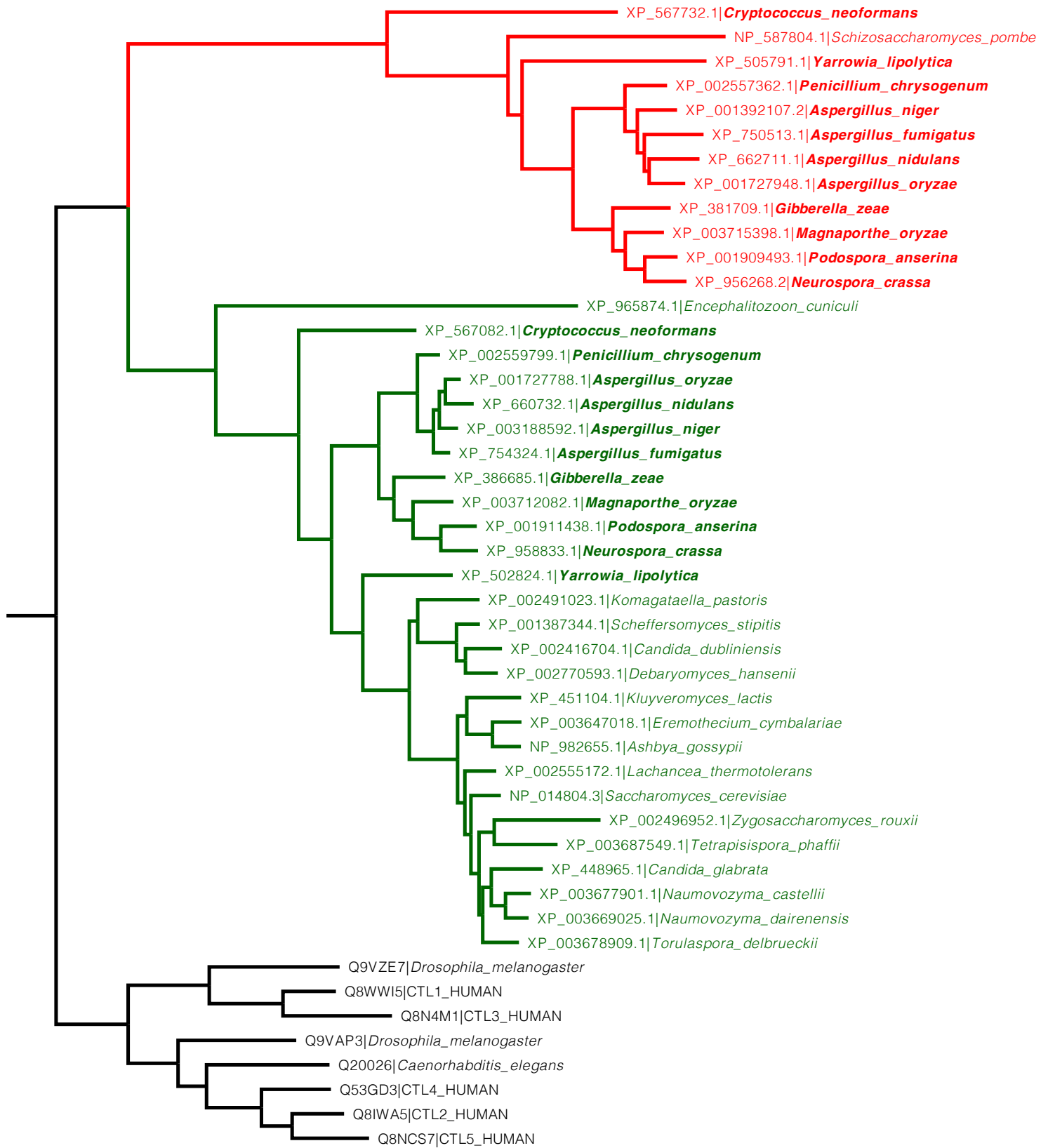

Supplement: Figure S6 — Phylogenetic relationship between CTL family proteins in fungi. The CTL family proteins in 28 fungi species were identified by exhaustive search using PSI-BLAST at MPI Bioinformatics Toolkit web server [82]. Multiple sequence alignment was generated using MAFFT [83]. Phylogenetic tree was created with FastTree [84] and visualized using FigTree (http://tree.bio.ed.ac.uk/). CTL proteins from three metazoan species (human, C. elegans, and D. melanogaster) were used as outgroup for rooting the tree. Among the 39 fungal proteins, the ones showing closer relationship with fission yeast Ctl1 protein (NP_587804.1) are colored red; the ones showing closer relationship with budding yeast Pns1 protein (NP_014804.3) are colored green. The 11 species with two CTL proteins are marked by bold font. (PDF) [file pgen.1003715.s006.pdf]

**A**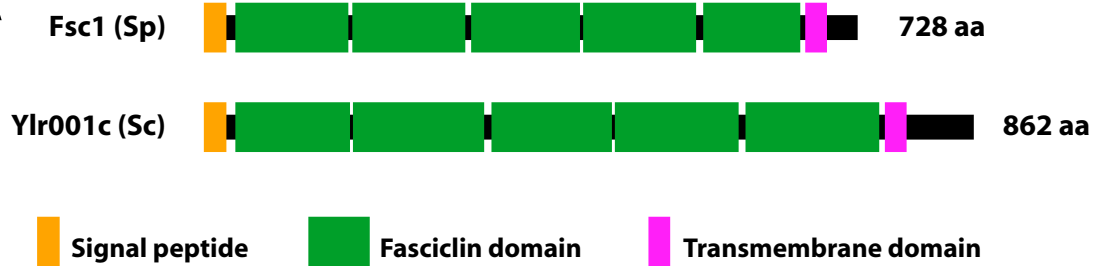**B**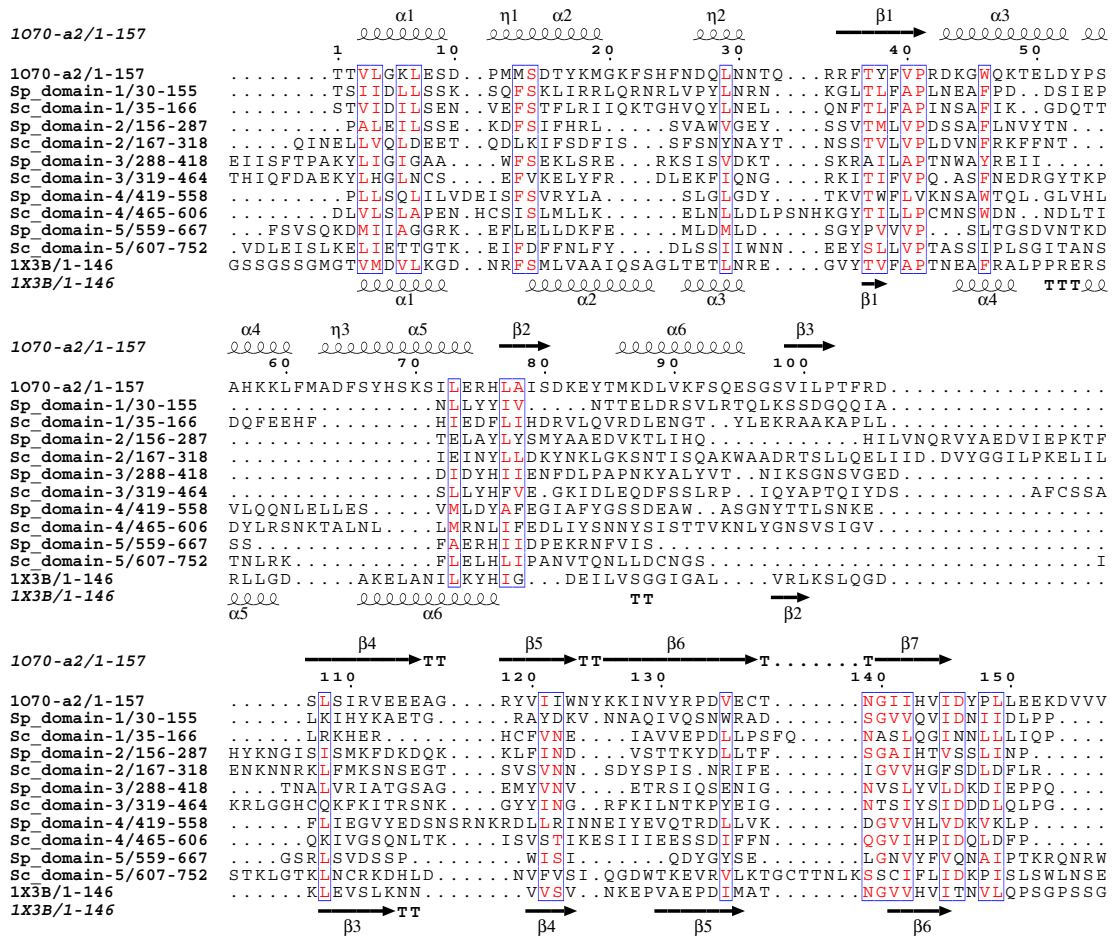

Supplement: Figure S7 — Fasciclin domains in S. pombe Fsc1 and S. cerevisiae Ylr001c. (A) The domain organizations of Fsc1 and Ylr001c. (B) The alignment of the individual fasciclin domains in Fsc1 and Ylr001c with two fasciclin domains whose 3D structures have been solved. The alignment was generated and edited with Jalview [85]. Secondary structural elements of the fourth fasciclin domain of Drosophila fasciclin I (PDB 1O70) and the fourth fasciclin domain of human transforming growth factor-beta-induced protein ig-h3 (PDB 1X3B) were visualized together with the sequence alignment using the ESPript web server (http://espript.ibcp.fr/) [86]. (PDF) [file pgen.1003715.s007.pdf]

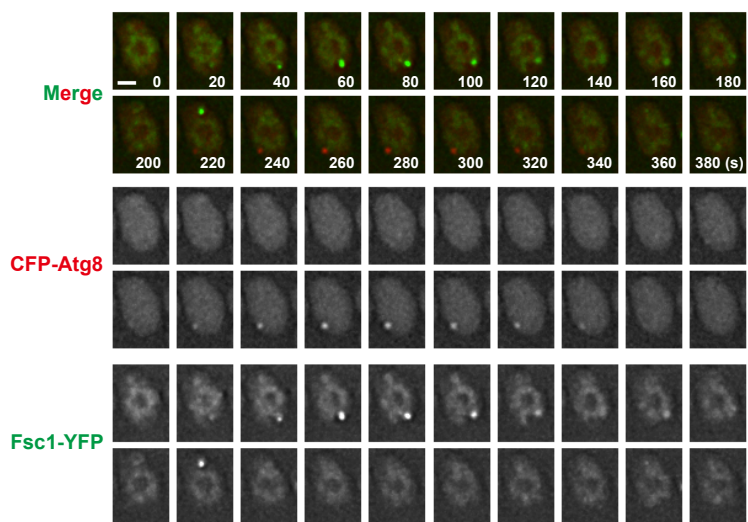

Supplement: Figure S8 — Time-lapse images of a cell expressing Fsc1-YFP and CFP-Atg8. Bar, 3 µm. (PDF) [file pgen.1003715.s008.pdf]

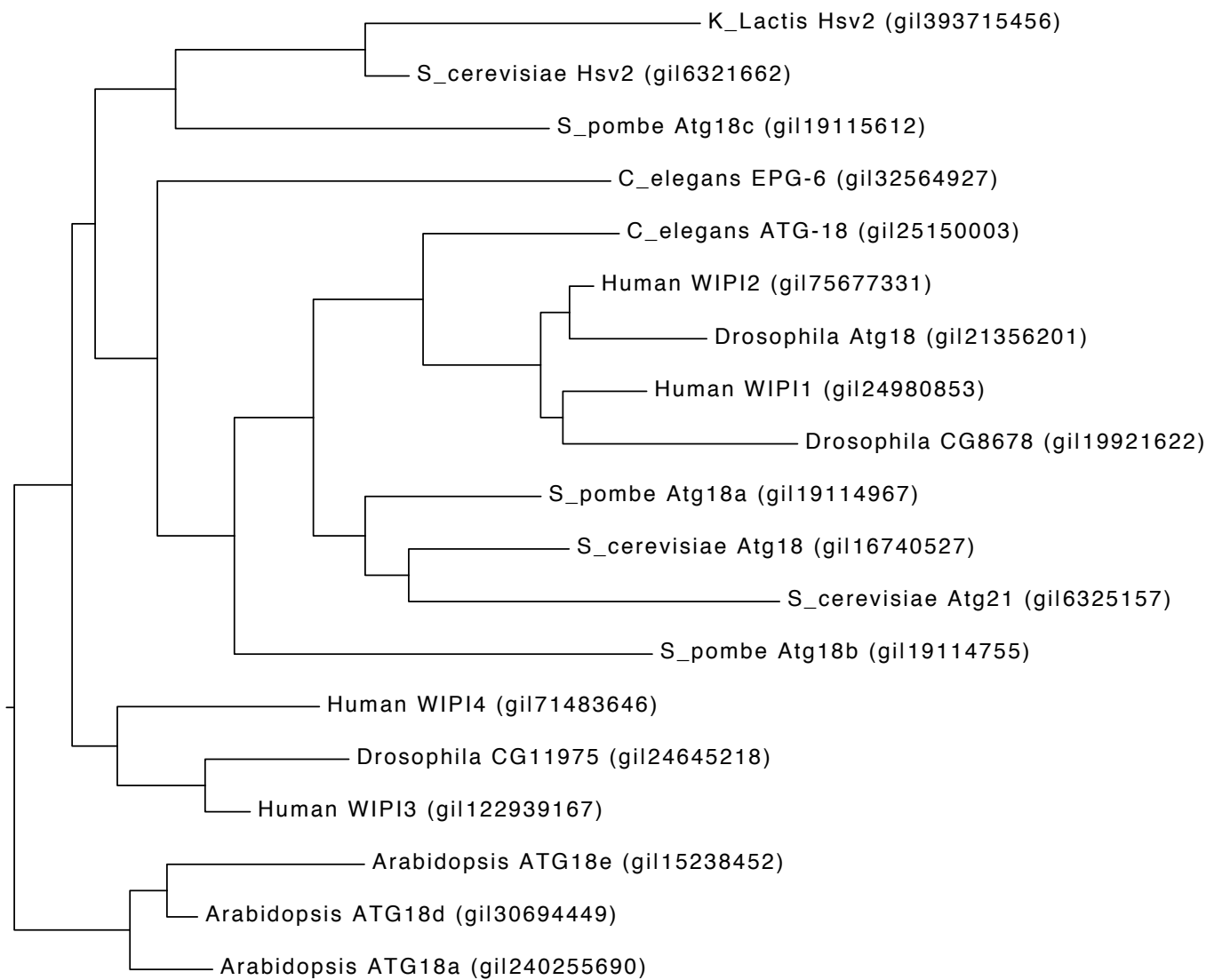

Supplement: Figure S10 — Phylogenetic relationship between Atg18/WIPI proteins. The sequence alignment in Figure S9 was used for phylogenetic tree construction. The phylogenetic tree was created with FastTree [84] and visualized using FigTree (http://tree.bio.ed.ac.uk/). Atg18 homologs from Arabidopsis were used as outgroup for rooting the tree. (PDF) [file pgen.1003715.s010.pdf]
