# Supplementary material for: Global Analysis of Fission Yeast Mating Genes Reveals New Autophagy Factors
Source: PLoS Genet. 2013 Aug 8;9(8):e1003715. doi: 10.1371/journal.pgen.1003715 (PMC3738441; doi:10.1371/journal.pgen.1003715)
Supplement: Figure S3 — Fission yeast SPAC227.04 protein shares homology with Atg10 proteins in other species. Genbank accession numbers are gi|18594496 (Homo sapiens), gi|161076388 (Drosophila melanogaster), gi|71984851 (Caenorhabditis elegans), gi|30680332 (Arabidopsis thaliana), gi|19113870 (Schizosaccharomyces pombe), and gi|6322986 (Saccharomyces cerevisiae). Red arrowhead points to the catalytic cysteine. Black arrowheads point to the two residues suggested to play critical roles in catalysis [78]. (PDF) [file pgen.1003715.s003.pdf]

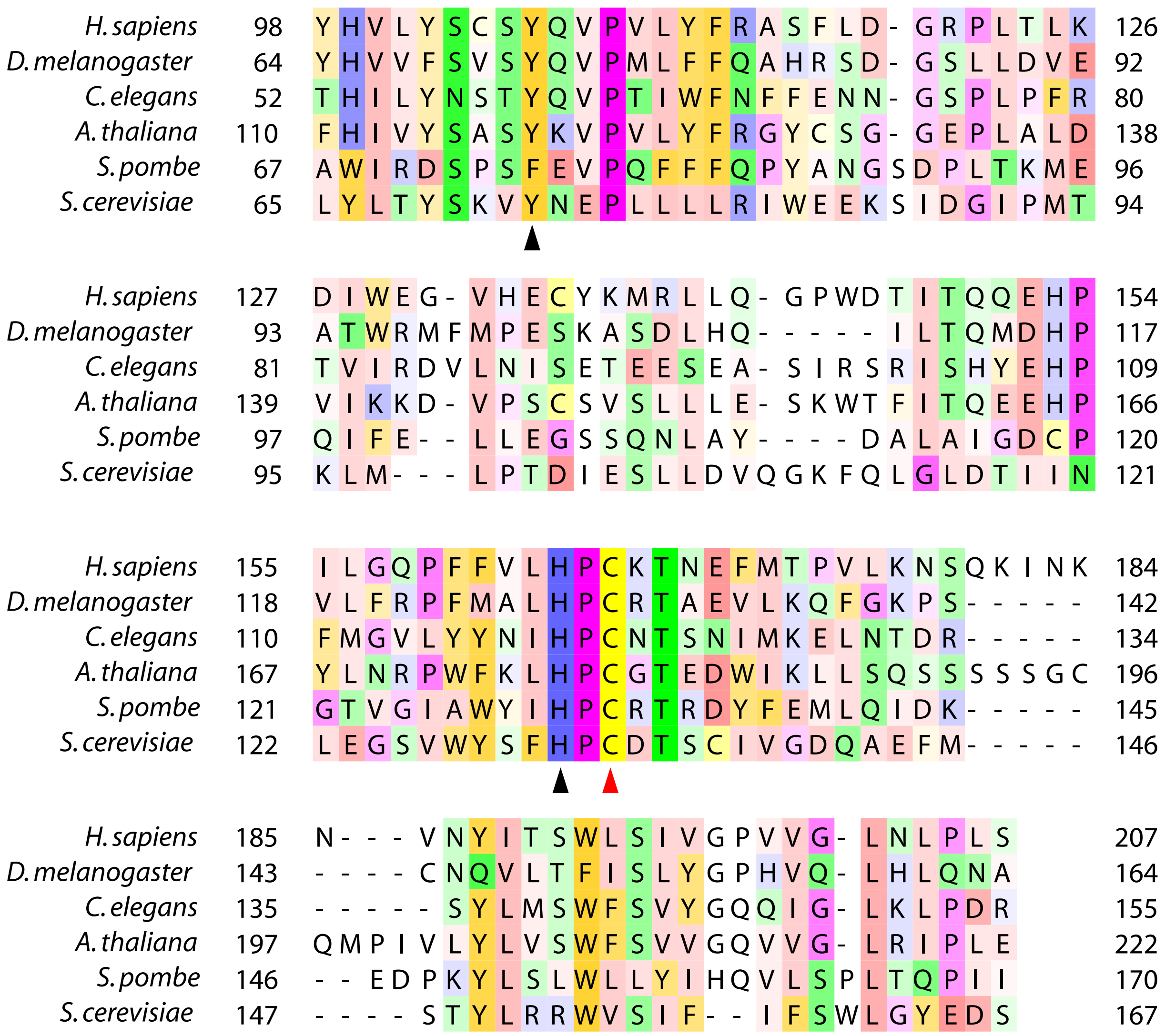
H. sapiens 98 YHVLYSCSYQVPVLYFRASFLD-GRPLTLK 126
D. melanogaster 64 YHVVFSVSYQVPMLFFQAHRSD-GSLLDVE 92
C. elegans 52 THILYNSTYQVPTIWFNFFENN-GSPLPFR 80
A. thaliana 110 FHIVYSASYKVPVLYFRGYCSG-GEPLALD 138
S. pombe 67 AWIRDSPSFEVPQFFFQPYANGSDPLTKME 96
S. cerevisiae 65 LYLTYSKVYNEPLLLLRIWEEKSIDGIPMT 94
H. sapiens 127 DIWEG-VHECYKMRLLQ-GPWDTITQQEHP 154
D. melanogaster 93 ATWRMFMPESKASDLHQ-----ILTQMDHP 117
C. elegans 81 TVIRDVLNISETEESEA-SIRSRISHYEHP 109
A. thaliana 139 VIKKD-VPSCSVSLLLE-SKWTFITQEEHP 166
S. pombe 97 QIFE--LLEGSSQNLAY----DALAIGDCP 120
S. cerevisiae 95 KLM---LPTDIESLLDVQGKFQLGLDTIIN 121
H. sapiens 155 ILGQPFFVLHPCKTNEFMTPVLKNSQKINK 184
D. melanogaster 118 VLFRPFMALHPCRTAEVLKQFGKPS----- 142
C. elegans 110 FMGVLYYNIHPCNTSNIMKELNTDR----- 134
A. thaliana 167 YLNRPWFKLHPCGTEDWIKLLSQSSSSSGC 196
S. pombe 121 GTVGIAWYIHPCRTRDYFEMLQIDK----- 145
S. cerevisiae 122 LEGSVWYSFHPCDTSCIVGDQAEFM----- 146
H. sapiens 185 N---VNYITSWLSIVGPVVG-LNLPLS 207
D. melanogaster 143 ----CNQVLTFISLYGPHVQ-LHLQNA 164
C. elegans 135 -----SYLMSWFSVYGQQIG-LKLPDR 155
A. thaliana 197 QMPIVLYLVSWFSVVGQVVG-LRIPLE 222
S. pombe 146 --EDPKYLSLWLLYIHQVLSPLTQPII 170
S. cerevisiae 147 ----STYLRRWVSIF--IFSWLGYEDS 167
