# Supplementary material for: Global Analysis of Fission Yeast Mating Genes Reveals New Autophagy Factors
Source: PLoS Genet. 2013 Aug 8;9(8):e1003715. doi: 10.1371/journal.pgen.1003715 (PMC3738441; doi:10.1371/journal.pgen.1003715)
Supplement: Figure S4 — Fission yeast SPBC405.05 protein shares homology with Atg16 proteins in other species. (A) Multiple sequence alignment of the Atg5-binding domain in Atg16 proteins. Open arrowheads point to the two residues important for the interaction between Atg5 and Atg16 in S. cerevisiae [79]. (B) Multiple sequence alignment of the coiled-coil domain (CCD) in Atg16 proteins. Filled arrowheads point to the four residues important for autophagic activity in S. cerevisiae [80]. (C) The domain organization of S. cerevisiae Atg16 protein (ScAtg16) and S. pombe Atg16 protein (SpAtg16). The domain boundaries of ScAtg16 is according to structural analysis [80]. The position of Atg5-binding domain in SpAtg16 is according to the alignment in (A). The position of CCD in SpAtg16 is as predicted by Marcoil using a probability threshold of 50% [81]. Genbank accession numbers are gi|124256480 (Homo sapiens), gi|62955681 (Danio rerio), gi|260796567 (Branchiostoma floridae), gi|198422508 (Ciona intestinalis), gi|28572018 (Drosophila melanogaster), gi|134117369 (Cryptococcus neoformans), gi|169844388 (Coprinopsis cinerea), gi|169625684 (Phaeosphaeria nodorum), gi|67515617 (Aspergillus nidulans), gi|19113100 (Schizosaccharomyces pombe), and gi|2497167 (Saccharomyces cerevisiae). (PDF) [file pgen.1003715.s004.pdf]

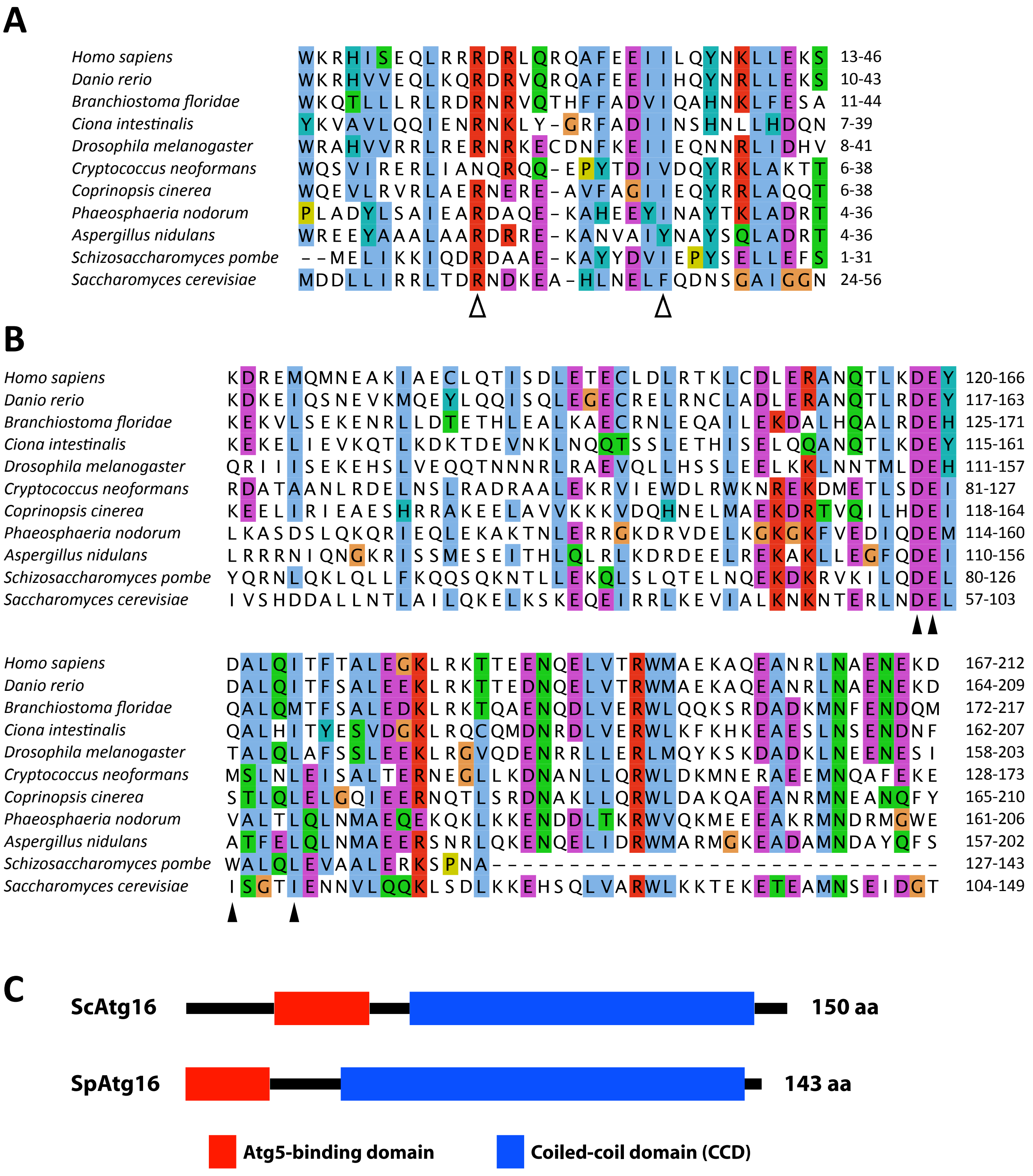
A
Homo sapiens WKRHISEQLRRRDRLQRQAFEEIILQYNKLLEKS 13-46
Danio rerio WKRHVVEQLKQRDRVQRQAFEEIIHQYNRLLEKS 10-43
Branchiostoma floridae WKQTLLLRLRDRNRVQTHFFADVIQAHNKLFESA 11-44
Ciona intestinalis YKVAVLQQIENRNKLY-GRFADIINSHNLLHDQN 7-39
Drosophila melanogaster WRAHVVRRLRERNRKECDNFKEIIEQNNRLIDHV 8-41
Cryptococcus neoformans WQSVIRERLIANQRQQ-EPYTDIVDQYRKLAKTT 6-38
Coprinopsis cinerea WQEVLRVRLAERNERE-AVFAGIIEQYRRLAQQT 6-38
Phaeosphaeria nodorum PLADYLSAIEARDAQE-KAHEEYINAYTKLADRT 4-36
Aspergillus nidulans WREEYAAALAARDRRE-KANVAIYNAYSQLADRT 4-36
Schizosaccharomyces pombe --MELIKKIQDRDAAE-KAYYDVIEPYSELLEFS 1-31
Saccharomyces cerevisiae MDDLLIRRLTDRNDKEA-HLNELFQDNSGAIGGN 24-56
B
Homo sapiens KDREMQMNEAKIAECLQTISDLETECLDLRTKLCDLERANQTLKDEY 120-166
Danio rerio KDKEIQSNEVKMQEYLQQISQLEGECRELRNCLADLERANQTLRDEY 117-163
Branchiostoma floridae KEKVLSEKENRLLDTETHLEALKAECRNLEQAILEKDALHQALRDEH 125-171
Ciona intestinalis KEKELIEVKQTLKDKTDEVNKLNQQTSSLETHISELQQANQTLKDEY 115-161
Drosophila melanogaster QRIIISEKEHSLVEQQTNNNRLRAEVQLLHSSLEELKKLNNTMLDEH 111-157
Cryptococcus neoformans RDATAANLRDELNSLRADRAALEKRVIEWDLRWKNREKDMETLSDEI 81-127
Coprinopsis cinerea KEELIRIEAESHRRAKEELAVVKKKVDQHNELMAEKDRTVQILHDEI 118-164
Phaeosphaeria nodorum LKASDSLQKQRIEQLEKAKTNLERRGKDRVDELKGKGKFVEDIQDEM 114-160
Aspergillus nidulans LRRRNIQNGKRISSMESEITHLQLRLKDRDEELREKAKLLEGFQDEI 110-156
Schizosaccharomyces pombe YQRNLQKLQLLFKQQSQKNTLLEKQLSLQTELNQEKDKRVKILQDEL 80-126
Saccharomyces cerevisiae IVSHDDALLNTLAILQKELKSKEQEIRRLKEVIALKNKNTERLNDEL 57-103
Homo sapiens DALQITFTALEGKLRKTTEENQELVTRWMAEKAQEANRLNAENEKD 167-212
Danio rerio DALQITFSALEEKLRKTTEDNQELVTRWMAEKAQEANRLNAENEKD 164-209
Branchiostoma floridae QALQMTFSALEDKLRKTQAENQDLVERWLQQKSRDADKMNFENDQM 172-217
Ciona intestinalis QALHITYESVDGKLRQCQMDNRDLVERWLKFKHKEAESLNSENDNF 162-207
Drosophila melanogaster TALQLAFSSLEEKLRGVQDENRRLLERLMQYKSKDADKLNEENESI 158-203
Cryptococcus neoformans MSLNLEISALTERNEGLLKDNANLLQRWLDKMNERAEEMNQAFEKE 128-173
Coprinopsis cinerea STLQLELGQIEERNQTLSRDNAKLLQRWLDAKQAEANRMNEANQFY 165-210
Phaeosphaeria nodorum VALTLQLNMAEQEKQKLKKENDDLTKRWVQKMEEEAKRMNDRMGWE 161-206
Aspergillus nidulans ATFELQLNMAEERSNRLQKENQELIDRWMARMGKEADAMNDAYQFS 157-202
Schizosaccharomyces pombe WALQLEVAALERKSPNA----------------------------- 127-143
Saccharomyces cerevisiae ISGTIENNVLQQKLSDLKKEHSQLVARWLKKTEKETEAMNSEIDGT 104-149
C
ScAtg16
150 aa
SpAtg16
143 aa
Atg5-binding domain
Coiled-coil domain (CCD)
