# Supplementary material for: Global Analysis of Fission Yeast Mating Genes Reveals New Autophagy Factors
Source: PLoS Genet. 2013 Aug 8;9(8):e1003715. doi: 10.1371/journal.pgen.1003715 (PMC3738441; doi:10.1371/journal.pgen.1003715)
Supplement: Figure S9 — The sequence alignment of Atg18/WIPI proteins. The alignment was generated and edited with Jalview [85]. Secondary structural elements of K. lactis Hsv2 (PDB 4EXV) were visualized together with the sequence alignment using the ESPript web server (http://espript.ibcp.fr/) [86]. The red bar denotes the FRRG motif involved in PI3P binding. The green and blue bars denote two sets of residues that are important for the Atg18-Atg2 interaction in S. cerevisiae, locating at the BC loop of blade 2 [87], and the loop connecting blade 2 and blade 3 [88], respectively. Genbank accession numbers of these proteins are listed in Figure S10. (PDF) [file pgen.1003715.s009.pdf]

```
4EXV/17-339                     β1  β2  β3  β4
                                1  10  20  30
4EXV/17-339                     IVDYEFNQDQSCLIVSTPKS.FDIY...NVHP.LKRIMSQE.............................
S_cerevisiae|Hsv2/19-448        FLSVSFNQDDSCFSVALENG.FRIF...NTDPLTSKLSKTF.............................
S_pombe|Atg18c/4-359            INTVSLNQDASCMSVALDTG.YKIF...QINP.L.KLRAQRQF...........................
C_elegans|EPG-6/36-375          LNHASVTLDHSAFAIADKDG.FKMY...QLNPLHFRMYKDY.............................
C_elegans|ATG-18/13-367         INYIGFNQDSKVICVGHKDG.YMFYK..TADILENNTLTYEGENLTHLGL....................
Human|WIPI2/15-359              LLFANFNQDNTSLAVGSKSG.YKFFSLSSVDK.LEQIYECT.............................
Drosophila|Atg18/14-366         EVFVNFNQNITSLAVATSGG.YSLYSLGSVDSTLDKIYHTK.............................
Human|WIPI1/17-361              LSCFSFNQDCTSLAIGTKAG.YKLFSLSSVEQ.LDQVHGSN.............................
Drosophila|CG8678/3-370         TYQMNFNQDFTSLSVLSPTG.LRLFSISSQDK.VEEIFAKD.............................
S_pombe|Atg18a/3-370            FFVRKYRGKAALLSIGTFDG.YKIY...NCDP.FGKCFHKI.............................
S_cerevisiae|Atg18/8-499        INFINFNQTGTCISLGTSKG.FKIF...NCEP.FGKFYSED.............................
S_cerevisiae|Atg21/1-496        MKVLQFNQDATCCVVAASSHQISIF...NCDP.FGKCFEIDTKNSKKKTSNNNGSASNSESRNNEESILI
S_pombe|Atg18b/5-335            ILYCSWNQDRGFLSIGSENG.YQVY...RSNP.FTLCFSKK.............................
Human|WIPI4/9-353               VTSLRFNQDQSCFCCAMETG.VRIY...NVEP.L.MEKGHLDH...........................
Drosophila|CG11975/13-334       LLYAAFNQDQGCFACATDTG.FRVY...NCDP.LKEKERQY.............................
Human|WIPI3/13-338              LLYAGFNQDHGCFACGMENG.FRVY...NTDP.LKEKEKQE.............................
Arabidopsis|ATG18e/33-362       VLSVAWNQVCSGFIVGTNHG.FNVY...SCKP.MIKKSISRAP...........................
Arabidopsis|ATG18d/36-386       LVSVSWNQDYSCFAAGTSHG.FRIY...NCEP.FKETFRRE.............................
Arabidopsis|ATG18a/78-420       VLHLSFNQDHACFAVGTDRG.FRIL...NCDP.FREIFRRD.............................

4EXV/17-339                     TT β5 TT β6  β7
                                40  50  60
4EXV/17-339                     ...........................MPDAGTIRMLHRTNYIAFVSTK.......KELLHIWDDV....
S_cerevisiae|Hsv2/19-448        ..................KESATNQSRGTGIGYTRMLYRTNYIALVGGG.KRPRHALNKLIIWDDL....
S_pombe|Atg18c/4-359            ..........................NDGGLSIVKMLFRSNVLLLVGGG.GNPKYAPNKLIVWDDV....
C_elegans|EPG-6/36-375          ........................VIKVGPVRLVKQDGNSRRIIYVSAL.AGGRFAQNNLMIFDVA....
C_elegans|ATG-18/13-367         ............................NNCLIIERLFSSALMVVISQ......KDPRVLHVYHFT....
Human|WIPI2/15-359              ...........................DTEDVCIVERLFSSSLVAIVSLK.....APRKLKVCHFK....
Drosophila|Atg18/14-366         ............................SDELFLIERLFESSLVAIVSQR.....APRKLKVCHFK....
Human|WIPI1/17-361              ...........................EIPDVYIVERLFSSSLVVVVSHT.....KPRQMNVYHFK....
Drosophila|CG8678/3-370         ...........................NTEQIRIVERLFNSSLVVLVTAQ.....KPNCLKMLHFK....
S_pombe|Atg18a/3-370            ...........................QGATSIVEMLFSTSLVALVE....KDDGNNRKLKLINTK....
S_cerevisiae|Atg18/8-499        ...........................SGGYAIVEMLFSTSLLALVGIG.DQPALSPRRLRIINTK....
S_cerevisiae|Atg21/1-496        TNGSRDRTDAEEEEDNEDNALVTGNILKEGEFVIEMLFSTSLIAIADRG..QGLNKGKKLKIVNTK....
S_pombe|Atg18b/5-335            ...........................ANGASICEMLYESSLLAFVNIS...PE.STRLLKLVDIK....
Human|WIPI4/9-353               .........................EQVGSMGLVEMLHRSNLLALVGGG.SSPKFSEISVLIWDDAREGK
Drosophila|CG11975/13-334       .........................FPEGGLSHVEMLFRCNYLALVGGG.IRPLYPPNKVIVWDDL....
Human|WIPI3/13-338              .........................FLEGGVGHVEMLFRCNYLALVGGG.KKPKYPPNKVMIWDDL....
Arabidopsis|ATG18e/33-362       ..........................HESGFKVAEMLFLSNLFAFVGNGYNNSEYPPNKVFVWDDY....
Arabidopsis|ATG18d/36-386       .........................LKDGGFKIVEMLFRSNILALVGGG.PNSQYPSNKVLIWDDH....
Arabidopsis|ATG18a/78-420       ........................FDRGGGVAVVEMLFRCNILALVGGG.PDPQYPPNKVMIWDDH....

4EXV/17-339                     β8  β9 TT β10 T.T β11  TT  β12
                                70  80  90  100  110
4EXV/17-339                     ..KKQDITRVKLDA...AVKDLFLSREFIVVSQG.DVISIFKFGNPWNKITEDIKF..............
S_cerevisiae|Hsv2/19-448        ..LQKETITLKFMS...SIKDVFLSRIHIVVVLE.NTIEIFQFQTNPQRICPILDI...PP.........
S_pombe|Atg18c/4-359            ..KERPVKELELNF...EIKGICFDGKLLAIATA.SKLFLYQFGNNLKLQRCLDTQ...NP.........
C_elegans|EPG-6/36-375          ..RNEEYFEITTPSRYGPITNIHVSPNRLVALNP.NRMFVWTYPDDIKQIRSEDIR.S.NP.........
C_elegans|ATG-18/13-367         ..SRNIICDHRFNK...SVLTVRLNRDRIVVCLE.DCIYIYNL.KDMKMMHNIMDT.PTNK.........
Human|WIPI2/15-359              ..KGTEICNYSYSN...TILAVKLNRQRLIVCLE.ESLYIHNI.RDMKVLHTIRET.PPNP.........
Drosophila|Atg18/14-366         ..KQSEICNYSYAN...TILAVKLNRERLIVCLE.ESLYIHNI.QDMKVVHTIRDT.PCNP.........
Human|WIPI1/17-361              ..KGTEICNYSYSS...NILSIRLNRQRLLVCLE.ESIYIHNI.KDMKLLKTLLDI.PANP.........
Drosophila|CG8678/3-370         ..KKQDICNCFYPS...EILCVRMNRQRLIVCLA.ESIHIHDI.RDMKILHSIENIAP.NE.........
S_pombe|Atg18a/3-370            ..KSTTICELTFPT...PLLAVKLNRKRLLAVLE.EQIYVYDI.SNMLLLHTIETT.S.NV.........
S_cerevisiae|Atg18/8-499        ..KHSIICEVTFPT...SILSVKMNKSRLVVLLQ.EQIYIYDI.NTMRLLHTIETN.P.NP.........
S_cerevisiae|Atg21/1-496        ..RKCTICEIVFPH...EIVDVVMNRKRMCVLLESDQIFIYDI.SCMKPLETIDLW...EDHYKRSQANS
S_pombe|Atg18b/5-335            ..RDIVLCRIFYPS...PVLSVRFTWNRLVVLIK.GSIYVYNL.KNMELINTLNTS..............
Human|WIPI4/9-353               DSKEKLVLEFTFTK...PVLSVRMRHDKIVIVLK.NRIYVYSFPDNPRKLFEFDTR.D.NP.........
Drosophila|CG11975/13-334       ..KKSPAISLDFNQ...PVRAVRLRRDRIVVVLE.GVIKVFTFTQQPQQLHVFETS.S.NP.........
Human|WIPI3/13-338              ..KKKTVIEIEFST...EVKAVKLRRDRIVVVLD.SMIKVFTFTHNPHQLHVFETC.Y.NP.........
Arabidopsis|ATG18e/33-362       ..RNCCLSELTFKS...EVIAVKLAREHVVVVLK.QNIYVYTF.NNLKVDRVIETL.M.NP.........
Arabidopsis|ATG18d/36-386       ..QGRCISEFTFRS...EIRAVKLRRDRIVVVLE.HKIYVYNF.MDLRLLHQIENM.A.NP.........
Arabidopsis|ATG18a/78-420       ..QGRCIGELSFRS...DVRSVRLRRDRIIVVLE.QKIFVYNF.SDLKLMHQIETI.A.NP.........

4EXV/17-339                     β13 TT
                                120
4EXV/17-339                     ...................................................GG.VCEFANG.........
S_cerevisiae|Hsv2/19-448        ...................................................NG.SVDYVVCSSKHLQSQA
S_pombe|Atg18c/4-359            ...................................................KG.LCAMVTT.........
C_elegans|EPG-6/36-375          ...................................................KG.ISAMSYD.........
C_elegans|ATG-18/13-367         ...................................................LG.VLDLTSNP........
Human|WIPI2/15-359              ...................................................AG.LCALSIN.........
Drosophila|Atg18/14-366         ...................................................QG.LCALSSS.........
Human|WIPI1/17-361              ...................................................TG.LCALSINH........
Drosophila|CG8678/3-370         ...................................................QG.LCALSLN.........
S_pombe|Atg18a/3-370            ...................................................FA.VCALSPN.........
S_cerevisiae|Atg18/8-499        ...................................................RG.LMAMSPS.........
S_cerevisiae|Atg21/1-496        FSNASNTGTLEGDSANLNRVATNLLANATQKSVNGSNPSVRTRRNSLRSKIRPRMVLSNDDR........
S_pombe|Atg18b/5-335            ...................................................KGNVIAFAVH.........
Human|WIPI4/9-353               ...................................................KG.LCDLCPS.........
Drosophila|CG11975/13-334       ...................................................NG.LCVLCPH.........
Human|WIPI3/13-338              ...................................................KG.LCVLCPN.........
Arabidopsis|ATG18e/33-362       ...................................................KG.LCCVTHV.........
Arabidopsis|ATG18d/36-386       ...................................................RG.LCCLSHH.........
Arabidopsis|ATG18a/78-420       ...................................................KG.LCAVSQG.........
```

```
                                   β14
4EXV/17-339                                 T..........................................................
                                   130

4EXV/17-339                    .........LLVYSNEFNL...................................................
S_cerevisiae|Hsv2/19-448       SQSQSKILEIIAFPSNKCV...................................................
S_pombe|Atg18c/4-359           .....VEKTAIVFP.SRKV...................................................
C_elegans|EPG-6/36-375         ...PTTAACYLAYP.GFKT...................................................
C_elegans|ATG-18/13-367        ......GNALIAYPGSTDT...................................................
Human|WIPI2/15-359             .....NDNCYLAYPGSATI...................................................
Drosophila|Atg18/14-366        .....SEHCYLAYPGSVTA...................................................
Human|WIPI1/17-361             ......SNSYLAYPGSLTS...................................................
Drosophila|CG8678/3-370        ........SHLAFPVCQTS...................................................
S_pombe|Atg18a/3-370           .....SENCYLAYPDSRDHEPRTEGESSS.........................................
S_cerevisiae|Atg18/8-499       .....VANSYLVYPSPPKVINSEIKAHATTNNITLSVGGNTETSFKRDQQDAGHSDISDLDQYSSFTKRD
S_cerevisiae|Atg21/1-496       ........SILCFTAYSSPKKNKPNSEAL.........................................
S_pombe|Atg18b/5-335           ......EN.YVAYNSPTNP...................................................
Human|WIPI4/9-353              .....LEKQLLVFP.GHKC...................................................
Drosophila|CG11975/13-334      .....SNKSLLAFP.GRRT...................................................
Human|WIPI3/13-338             .....SNNSLLAFP.GTHT...................................................
Arabidopsis|ATG18e/33-362      .....ESKAVLACP.GFHP...................................................
Arabidopsis|ATG18d/36-386      .....MNTSVLACPGIRR....................................................
Arabidopsis|ATG18a/78-420      .....VGSMVLVCPGLQK....................................................

                                                  β15                  β16       β17         β18
4EXV/17-339                    .................T                                           TT
                                                 140       150          160       170       180

4EXV/17-339                    .................GQIHVTRLQTDAEQVVGK.......GVLVKA.HANPVKMVRLNRKGDMVATCS
S_cerevisiae|Hsv2/19-448       .................GQIQVADLSQIKYNSQNPKESALLPTSIIKA.HKNPIKLVRLNRQGTMVATCS
S_pombe|Atg18c/4-359           .................GQLQILFLFKDHMN...........TSIVPA.HDSEISCLGISKTGSKIASSS
C_elegans|EPG-6/36-375         .................GSVQIMHLNALTARESKS.......PIVIEA.HLTDIAQVALNCQGTLVATGS
C_elegans|ATG-18/13-367        .................GSVHLFDAINLSS............VSTFNA.HEGTIACLKFNQEGNMIATAS
Human|WIPI2/15-359             .................GEVQVFDTINLRA............ANMIPA.HDSPLAALAFDASGTKLATAS
Drosophila|Atg18/14-366        .................GEVQIFDAINLHA............KTMIPA.HDTPLAALAFSPSGTEIATAS
Human|WIPI1/17-361             .................GEIVLYDGNSLKT............VCTIAA.HEGTLAAITFNASGSKLASAS
Drosophila|CG8678/3-370        .................GELRIFNASKLRT............GMTIRA.HDTSLSALAFSPSGALLATAS
S_pombe|Atg18a/3-370           ........PNVSNSAVSGQVILWDVINCKQ............ITKIEA.HKDSLACLAFNSDGTMLATAS
S_cerevisiae|Atg18/8-499       DADPTSSNGGNSSIIKNGDVIVFNLETLQP............TMVIEA.HKGEIAAMAISFDGTLMATAS
S_cerevisiae|Atg21/1-496       .................YDVVIYDTLNVTP............VNYLNSVHKGNVACLAVSHDGKLLATAS
S_pombe|Atg18b/5-335           .................GDIYLASLDTAIP............VTLIHC.HSSAVQVVDFHPRGHLIATAS
Human|WIPI4/9-353              .................GSLQLVDLASTKPGTSSA.......PFTINA.HQSDIACVSLNQPGTVVASAS
Drosophila|CG11975/13-334      .................GHVQIVDLANTERA...........PLEVIA.HEAGISCIALNLQGTRLATAG
Human|WIPI3/13-338             .................GHVQLVDLASTEKP...........PVDIPA.HEGVLSCIALNLQGTRIATAS
Arabidopsis|ATG18e/33-362      .................GQVQVHDLRWNV.............IKFIKA.HDSAIACMTLTLDGSLLATAS
Arabidopsis|ATG18d/36-386      .................GEVRVEHFGLNM.............VQIINA.HDSNIACMTLTLDGLLLATAS
Arabidopsis|ATG18a/78-420      .................GQVRIEHYASKR.............TKFVMA.HDSRIACFALTQDGHLLATAS

                                    β19                β20            β21         β22        β23
4EXV/17-339                    TT                                         TT
                                  190                 200       210       220       230

4EXV/17-339                    QDGTLIRVFQT.......DNGVLVREFRRGLD.RTSIIDMRWSPDGSKLAVVSDKWTLHVFEV.......
S_cerevisiae|Hsv2/19-448       VQGTLIRIFST.......HNGTLIKEFRRGVD.KADIYEMSFSPNGSKLAVLSNKQTLHIFQI.......
S_pombe|Atg18c/4-359           TNGTLIRIWNS.......ETGEKICEFRRGYQ.HTAVCQLAFSPDELLLACASKKETLHIFSLHGSPNTI
C_elegans|EPG-6/36-375         TKGTVIRVFDA.......RTKGPLYELRRGTV.QAHLQCMAFSPCSSYLAVASDKGTLHMFGI.......
C_elegans|ATG-18/13-367        TKGTVIRVYSV.......PNGHRLFEFRRGVTRCVNIYSLCFSSDSKYLTSSSNTETVHVFKL.......
Human|WIPI2/15-359             EKGTVIRVFSI.......PEGQKLFEFRRGVKRCVSICSLAFSMDGMFLSASSNTETVHIFKL.......
Drosophila|Atg18/14-366        ERGTVIRVFSS.......QDGSRLFELRRGLKRCVSIVSLSFSTCAEYLVSSSNTETVHIFRLDRSATET
Human|WIPI1/17-361             EKGTVIRVFSV.......PDGQKLYEFRRGMKRYVTISSLVFSMDSQFLCASSNTETVHIFKL.......
Drosophila|CG8678/3-370        ERGTVIRVFCV.......KNGQRVQEFRRGVS.CVRIASLVFSASGDFLCASSNTETVHVFKIDTRAVET
S_pombe|Atg18a/3-370           DNGRIIRVFAI.......PSGQRLYQFRRGSL.PAQIYSIAFHPDSSLLTVTSSTQTVHIFRL.KEVYSN
S_cerevisiae|Atg18/8-499       DKGTIIRVFDI.......ETGDKIYQFRRGTY.ATRIYSISFSEDSQYLAVTGSSKTVHIFKLGHSMSNN
S_cerevisiae|Atg21/1-496       DKGTIIRVFHTGVDSDYMSSRSLFKEFRRGTR.LCNLYQLAFDKSMTMIGCVGDTDTIHLFKLDDASNSL
S_pombe|Atg18b/5-335           AKGTVIRVITT.......SDGELVTELRRGYI.PASIVSISFHPVEPFLACASENGTIHVFKISKQPSDP
Human|WIPI4/9-353              QKGTLIRLFDT.......QSKEKLVELRRGTD.PATLYCINFSHDSSFLCASSDKGTVHIFAL.......
Drosophila|CG11975/13-334      EKGTLIRIFDT.......ESGKKVSELRRGSN.HANIFCINFNHQSTMVVVASDHGTIHVFNL.......
Human|WIPI3/13-338             EKGTLIRIFDT.......SSGHLIQELRRGSQ.AANIYCINFNQDASLICVSSDHGTVHIFAA.......
Arabidopsis|ATG18e/33-362      TKGTLIRIFNA.......VDGTLLQEFRRGVE.RAEIYNVAISNLKWVAASSEKGTLHVFRL.......
Arabidopsis|ATG18d/36-386      TKGTLIRIFNT.......MDGTRLQEVRRGVD.RADIYSIALSPNVQWLAVSSDKGTVHIFSLRVRVIGE
Arabidopsis|ATG18a/78-420      SKGTLVRIFNT.......VDGTLRQEVRRGAD.RAEIYSLAFSSNAQWLAVSSDKGTVHVFGL....KV

                                                                                      α1
4EXV/17-339                                                                                  T.
                                                                                   240

4EXV/17-339                    .........................................................FNDAENKRHVLK.
S_cerevisiae|Hsv2/19-448       .............................................FETTNTETNTPDHSRANGSSHPLK.
S_pombe|Atg18c/4-359           RQLTSEEPYEEAS.....................................EFKSSTTEPRQTHWKRKLL.
C_elegans|EPG-6/36-375         ................................................RDAEPQKKKNVLERSRGSSSI.
C_elegans|ATG-18/13-367        .............................................EKTEGVDNKPEASTEGGGWFDAINK
Human|WIPI2/15-359             ..................................................ETVKEKPPEEPTTWTGYFGK
Drosophila|Atg18/14-366        AEGHGSKQ..........................................SSDDWMGYSFFRFLSKTVT.
Human|WIPI1/17-361             ..................................................EQVTNSRPEEPSTWSGYMGK
Drosophila|CG8678/3-370        VELKAIADVAAKSENSAKEAVA............................STAAEESCRPVASWGGMFSK
S_pombe|Atg18a/3-370           LERQGLLPSSPPPKE...................................SLLRRSS....RSLIGTVG.
S_cerevisiae|Atg18/8-499       KLDSDDSNMEEAAADDSSLDTTSIDALSDEENPTRLAREPYVDASRKTMGRMIRYSSQKLSRRAARTLG.
S_cerevisiae|Atg21/1-496       PGDNSSNGHWNEEEYILASNSNPSMGTP......................KEIPLSKPRIANYFSKKIK.
S_pombe|Atg18b/5-335           ..................................................NSSPTSSVTVSSSWSKYLT.
Human|WIPI4/9-353              ...................................................KDTRLNRRSALARVGKVG.
Drosophila|CG11975/13-334      ..................................................EDNKPRESSL..........
Human|WIPI3/13-338             ..................................................EDPKRNKQSSLASA......
Arabidopsis|ATG18e/33-362      ....................................................RPDILSFDPASSSSFIR.
Arabidopsis|ATG18d/36-386      DAYSTEHETSSNSLQ...................................PLVSPASGANPGSSLSFLR.
Arabidopsis|ATG18a/78-420      NSGSQVKDSS........................................RIAPDATPSSPSSSLSLFK.
```

```
                                                          β24                                 β25
4EXV/17-339                      ......T                                                      T.
                                      250       260       270                       280
4EXV/17-339                      ......DWIN.....IKYFQSEWS.....ICNFKLKVS..................KGSNDCKIAWISD.
S_cerevisiae|Hsv2/19-448         ......NYIPKGLWRPKYLDSVWS.....ICNAHLKNPIFDAHRNDNSGDVTHDNEFYKDRCRIGWCQD.
S_pombe|Atg18c/4-359             ......KLID......SGKRAHWR..........IQLY...................QSNPVLLHWLDE.
C_elegans|EPG-6/36-375           ..................VKIQLDRPVMAIGFGKIPET..................PKNLQSIIAIC...
C_elegans|ATG-18/13-367          TFS...AYMP..SQVLQ.VGELMT.TERSFATAKLPGA.................ARSNQVSLVSHKNQ.
Human|WIPI2/15-359               VLMASTSYLP......SQVTEMFN.QGRAFATVRLPFC..................GHKNICSLATIQK.
Drosophila|Atg18/14-366          ......SYLP......TQVTDVFS.QGRAFASVTLPEA..................GVRRMCAIATIQK.
Human|WIPI1/17-361               MFMAATNYLP......TQVSDMMH.QDRAFATARLNFS..................GQRNICTLSTIQK.
Drosophila|CG8678/3-370          AVS..SLLLP......TQVSEVLA.QDRSFATVQLAQG....................GLKHICALTRV.
S_pombe|Atg18a/3-370             ......GYLP......QSVSGMLD.PERDFAYAHIPGD....................KVTSIAAFGPD.
S_cerevisiae|Atg18/8-499         ......QIFP......IKVTSLLE.SSRHFASLKLPVETNSHVMTISSIGSPIDIDTSEYPELFETGNSA
S_cerevisiae|Atg21/1-496         ......SSIP.........NQNLS...RNFAYITVNES.....................NRSCLGFPDE.
S_pombe|Atg18b/5-335             ................SNVAKVWD.TRKEFATAKIPEA....................SFYGKIIFSSS.
Human|WIPI4/9-353                ......PMIG......QYVDSQWS.....LASFTVPAE.....................SACICAFGRN.
Drosophila|CG11975/13-334        ......PIIP......KYFSSQWS.....FVKFSIPQG.....................PRCVCAFGAD.
Human|WIPI3/13-338               ......SFLP......KYFSSKWS.....FSKFQVPSG.....................SPCICAFGTE.
Arabidopsis|ATG18e/33-362        ......VILP......KY...LYE.NERSFAQFSLPAS.....................TKFIVGFGSE.
Arabidopsis|ATG18d/36-386        ......GVLP......KYFSSEWS.....FSQFHVPEV.....................TQYFAAFGAQ.
Arabidopsis|ATG18a/78-420        ......GVLP......RYFSSEWS.....VAQFRLVEG.....................TQYIAAFGHQ.
```

```
                                              β26                     β27                           β28
4EXV/17-339                      .............T                ........................
                                               290       300                         310
4EXV/17-339                      ............TGLVIVWPNRRLADTFKL......................NYNDDE.....HVWWLQL
S_cerevisiae|Hsv2/19-448         .....SNNREQDDSLVLVWQNSGIWEKFVILEKEQQDSSKTHYSLNESLRNEDTKSAG...EPTRWELVR
S_pombe|Atg18c/4-359             ............MTILICY.KDAAYQKLKL......................TIEESSKSVEHANQHVCF
C_elegans|EPG-6/36-375           ....................ADATYWRHEF......................YKDNTG......NFTSHF
C_elegans|ATG-18/13-367          ............QYVMAAT.SDGFVYAYRL.......................DPEGG........ELDL
Human|WIPI2/15-359               ...........IPRLLVGA.ADGYLYMYNL......................DPQEGG........ECAL
Drosophila|Atg18/14-366          ...........QLRLLIAS.QDGYLYVYSI......................PTVEGA........ECQL
Human|WIPI1/17-361               ...........LPRLLVAS.SSGHLYMYNL......................DPQDGG........ECVL
Drosophila|CG8678/3-370          .........QKEPRLLIAC.EDGFLYVHEF......................PAERGG........PCKL
S_pombe|Atg18a/3-370             ............NTIVNVATYDGNLYSFRV......................NLRTGG........ECAM
S_cerevisiae|Atg18/8-499         STESYHEPVMKMVPIRVVS.SDGYLYNFVM......................DPERGG........DCLI
S_cerevisiae|Atg21/1-496         ..........FPNQVYIAS.DDGTFSIYSI.......................PSKPG........ECVL
S_pombe|Atg18b/5-335             ...........GPHIQVAS.YSGHYYRFAV......................NLKNGG........NCAL
Human|WIPI4/9-353                .......TSKNVNSVIAIC.VDGTFHKYVF........................TPDG........NCNR
Drosophila|CG11975/13-334        ...........PNSVVAIC.ADGHYYKFLF........................NNKG........ECSR
Human|WIPI3/13-338               ...........PNAVIAIC.ADGSYYKFLF........................NPKG........ECIR
Arabidopsis|ATG18e/33-362        ............NTVLLVG.IDGSFRRCKF......................DHADGG........QMVE
Arabidopsis|ATG18d/36-386        ............NTIAIIG.LDGSFYRCNF......................DPVNGG........EMTQ
Arabidopsis|ATG18a/78-420        ...........KNTVVILG.MDGSFYRCQF......................DPVNGG........EMSQ
```

```
4EXV/17-339
                                   320
4EXV/17-339                      NQRNEIPL
S_cerevisiae|Hsv2/19-448         ESWREL..
S_pombe|Atg18c/4-359             HYDYTLEA
C_elegans|EPG-6/36-375           GSYDELIE
C_elegans|ATG-18/13-367          IKQHNIGP
Human|WIPI2/15-359               MKQHRLDG
Drosophila|Atg18/14-366          IKRHDLRL
Human|WIPI1/17-361               IKTHSLLG
Drosophila|CG8678/3-370          MSVHDLRG
S_pombe|Atg18a/3-370             VNHFCVGL
S_cerevisiae|Atg18/8-499         LSQYSILM
S_cerevisiae|Atg21/1-496         TKNNKFT.
S_pombe|Atg18b/5-335             LERYIFDD
Human|WIPI4/9-353                EAFDVYLD
Drosophila|CG11975/13-334        DICTQFLE
Human|WIPI3/13-338               DVYAQFLE
Arabidopsis|ATG18e/33-362        LEHKYFFS
Arabidopsis|ATG18d/36-386        LEHFHFLK
Arabidopsis|ATG18a/78-420        LEYHNCLK
```
