# Supplementary material for: Global Analysis of Fission Yeast Mating Genes Reveals New Autophagy Factors
Source: PLoS Genet. 2013 Aug 8;9(8):e1003715. doi: 10.1371/journal.pgen.1003715 (PMC3738441; doi:10.1371/journal.pgen.1003715)
Supplement: Table S2 — The mating conditions of the 22 screens. (PDF) [file pgen.1003715.s012.pdf]

Table S2

Mating conditions of the 22 screens

	Screen name	Date	Pregrowth medium (liquid)	Type of mating medium (solid)	Concentration of glucose added into mating medium (w/v)	Concentration of supplements (leucine, adenine, and uracil) added into mating medium (mg/l)	Concentration of NH_4Cl added into mating medium (mM)	Mating temperature (℃)
	0428_YES_SPA-100s-30n	04-28-2010	YES	SPA	1%	100	30	25
	0428_YES_SPA-100s	04-28-2010	YES	SPA	1%	100	0	25
	0428_YES_SPA-200s	04-28-2010	YES	SPA	1%	200	0	25
*	0428_YES_SPA-45s	04-28-2010	YES	SPA	1%	45	0	25
	0521_EMM_EMM1g-45s	05-21-2010	supplemented EMM	EMM (1%glucose)	1%	45	93.5	25
	0521_EMM_EMMnoN-45s	05-21-2010	supplemented EMM	EMM-N	2%	45	0	25
	0521_EMM_EMM-45s	05-21-2010	supplemented EMM	EMM	2%	45	93.5	25
	0521_EMM_EMM-225s	05-21-2010	supplemented EMM	EMM	2%	225	93.5	25
	0521_EMM_SPA-200s	05-21-2010	supplemented EMM	SPA	1%	200	0	25
	0521_EMM_SPA-45s	05-21-2010	supplemented EMM	SPA	1%	45	0	25
	0521_EMM_YEPD	05-21-2010	supplemented EMM	YEPD	2%	0	0	25
	0521_YES_EMM1g-45s	05-21-2010	YES	EMM (1%glucose)	1%	45	93.5	25
	0521_YES_EMMnoN-45s	05-21-2010	YES	EMM-N	2%	45	0	25
	0521_YES_EMM-45s	05-21-2010	YES	EMM	2%	45	93.5	25
	0521_YES_EMM-225s	05-21-2010	YES	EMM	2%	225	93.5	25
	0521_YES_ME	05-21-2010	YES	ME (malt extract)	0%	0	0	25
	0521_YES_SPA-200s	05-21-2010	YES	SPA	1%	200	0	25
*	0521_YES_SPA-45s	05-21-2010	YES	SPA	1%	45	0	25
	0521_YES_YEPD	05-21-2010	YES	YEPD	2%	0	0	25
	1108_YES_SPA-45s_20d	11-08-2010	YES	SPA	1%	45	0	20
*	1108_YES_SPA-45s_25d	11-08-2010	YES	SPA	1%	45	0	25
	1108_YES_SPA-45s_30d	11-08-2010	YES	SPA	1%	45	0	30

* Standard mating conditions
