# Supplementary material for: Global Analysis of Fission Yeast Mating Genes Reveals New Autophagy Factors
Source: PLoS Genet. 2013 Aug 8;9(8):e1003715. doi: 10.1371/journal.pgen.1003715 (PMC3738441; doi:10.1371/journal.pgen.1003715)
Supplement: Table S4 — The strains used in this study. (PDF) [file pgen.1003715.s014.pdf]

Table S4. Strains used in this study

Strain	Genotype	
DY3984	h^-	*leu1-32 ura4-D18 ade6-M210 (or M216)*
DY11904	h^-	*CFP-atg8::leu1+ leu1-32*
DY11900	h^-	*atg1Δ::kanMX CFP-atg8::leu1+ leu1-32*
DY4019	h^-	*atg2Δ::kanMX CFP-atg8::leu1+ leu1-32*
DY11899	h^-	*atg3Δ::kanMX CFP-atg8::leu1+ leu1-32*
DY4003	h^-	*atg4Δ::kanMX CFP-atg8::leu1+ leu1-32*
DY4021	h^-	*atg5Δ::kanMX CFP-atg8::leu1+ leu1-32*
DY11898	h^-	*atg6Δ::kanMX CFP-atg8::leu1+ leu1-32*
DY4023	h^-	*atg7Δ::kanMX CFP-atg8::leu1+ leu1-32*
DY4025	h^-	*atg9Δ::kanMX CFP-atg8::leu1+ leu1-32*
DY4029	h^-	*atg13Δ::kanMX CFP-atg8::leu1+ leu1-32*
DY4027	h^-	*atg11Δ::kanMX CFP-atg8::leu1+ leu1-32*
DY4008	h^-	*atg17Δ::kanMX CFP-atg8::leu1+ leu1-32*
DY11877	*h?*	*atg101Δ::kanMX CFP-atg8::leu1+ leu1-32*
DY4133	*h?*	*isp6Δ::kanMX CFP-atg8::leu1+ leu1-32*
DY4031	h^-	*atg18aΔ::kanMX CFP-atg8::leu1+ leu1-32*
DY4012	h^-	*atg18cΔ::kanMX CFP-atg8::leu1+ leu1-32*
DY4010	h^-	*atg18bΔ::kanMX CFP-atg8::leu1+ leu1-32*
DY11930	h^-	*atg10Δ::kanMX CFP-atg8::leu1+ leu1-32*
DY11931	h^-	*atg14Δ::kanMX CFP-atg8::leu1+ leu1-32*
DY11932	h^-	*atg16Δ::kanMX CFP-atg8::leu1+ leu1-32*
DY11929	h^-	*ctl1Δ::kanMX CFP-atg8::leu1+ leu1-32*
DY11928	h^-	*fsc1Δ::kanMX CFP-atg8::leu1+ leu1-32*
DY11880	h^-	*atg15Δ::kanMX CFP-atg8::leu1+ leu1-32*
DY11901	h^-	*atg12Δ::kanMX CFP-atg8::leu1+ leu1-32*
DY11860	h^-	*spbc18h10.19hΔ::kanMX CFP-atg8::leu1+ leu1-32*
DY11876	h^-	*spcc417.09cΔ::kanMX CFP-atg8::leu1+ leu1-32*
DY11874	h^-	*spcc757.04Δ::kanMX CFP-atg8::leu1+ leu1-32*
DY11936	h^-	*atg5-TAP::natMX his3-D1 leu1-32*
DY11867	*h?*	*atg7Δ::kanMX atg5-TAP::natMX*
DY11862	*h?*	*atg10Δ::kanMX atg5-TAP::natMX*
DY11865	*h?*	*atg12Δ::kanMX atg5-TAP::natMX*
DY11863	*h?*	*atg14Δ::kanMX atg5-TAP::natMX*

DY11866	*h?*	*atg16Δ::kanMX atg5-TAP::natMX*
DY11935	h^{+}	*atg5-myc::kanMX his3-D1 leu1-32*
DY3963	h^{+}	*atg16-YFH::leu1+ his3-D1 leu1-32*
DY11869	*h?*	*atg16-YFH::leu1+ atg5-myc::natMX his3-D1 leu1-32*
DY11868	*h?*	*atg12Δ::kanMX atg16-YFH::leu1+ atg5-myc::natMX his3-D1 leu1-32*
DY11934	h^{-}	*atg14-myc::kantMX his3-D1 leu1-32*
DY3957	h^{+}	*atg6-YFH::leu1+ his3-D1 leu1-32*
DY11861	*h?*	*atg6-YFH::leu1+ atg14-myc::kantMX his3-D1 leu1-32*
DY11870	h^{+}	*atg2-YFH::leu1+ CFP-atg8::leu1+ leu1-32*
DY11871	*h?*	*atg18a-YFH::leu1+ CFP-atg8::leu1+ leu1-32*
DY11872	*h?*	*atg18b-YFH::leu1+ CFP-atg8::leu1+ leu1-32*
DY11875	*h?*	*atg18c-YFH::leu1+ CFP-atg8::leu1+ leu1-32*
DY3965	*h?*	*atg1-YFH::leu1+ CFP-atg8::leu1+ leu1-32*
DY3969	h^{+}	*atg5-YFH::leu1+ CFP-atg8::leu1+ leu1-32*
DY3970	*h?*	*atg11-YFH::leu1+ CFP-atg8::leu1+ leu1-32*
DY3976	h^{-}	*atg14-YFH::leu1+ CFP-atg8::leu1+ leu1-32*
DY3980	*h?*	*atg16-YFH::leu1+ CFP-atg8::leu1+ leu1-32*
DY11903	h^{-}	*ctl1-YFH::leu1+ CFP-atg8::leu1+ leu1-32*
DY11857	*h?*	*atg13-YFH::leu1+ CFP-atg8::leu1+ leu1-32*
DY11858	*h?*	*atg17-YFH::leu1+ CFP-atg8::leu1+ leu1-32*
DY11859	*h?*	*atg101-YFH::leu1+ CFP-atg8::leu1+ leu1-32*
DY3972	h^{+}	*atg6-YFH::leu1+ CFP-atg8::leu1+ leu1-32*
DY11905	h^{-}	*atg9-YFP::kanMX CFP-atg8::leu1+ leu1-32*
DY11893	*h?*	*atg1-YFH::leu1+ zhf1-mCherry::kanMX his3-D1 leu1-32*
DY11892	*h?*	*atg11-YFH::leu1+ zhf1-mCherry::kanMX his3-D1 leu1-32*
DY11891	*h?*	*atg18a-YFH::leu1+ zhf1-mCherry::kanMX his3-D1 leu1-32*
DY11889	*h?*	*atg18a-YFH::leu1+ hse1-mCherry::natMX CFP-atg8::leu1+ leu1-32*
DY11873	*h?*	*atg6-YFH::leu1+ vps32-mCherry::kanMX CFP-atg8::leu1+ leu1-32*
DY11914	*h?*	*atg18aΔ::kanMX atg2-YFH::leu1+ CFP-atg8::leu1+ leu1-32*
DY11913	*h?*	*atg18aΔ::kanMX atg1-YFH::leu1+ CFP-atg8::leu1+ leu1-32*
DY11925	*h?*	*atg18aΔ::kanMX atg16-YFH::leu1+ CFP-atg8::leu1+ leu1-32*
DY11916	*h?*	*atg18aΔ::kanMX atg14-YFH::leu1+ CFP-atg8::leu1+ leu1-32*
DY11927	*h?*	*atg18aΔ::kanMX atg13-YFH::leu1+ CFP-atg8::leu1+ leu1-32*
DY11915	*h?*	*atg18aΔ::kanMX atg5-YFH::leu1+ CFP-atg8::leu1+ leu1-32*
DY11864	*h?*	*atg18aΔ::kanMX atg5-TAP::natMX*
DY11883	*h?*	*atg18aΔ::kanMX atg16-YFH::leu1+ atg5-myc::natMX his3-D1 leu1-32*
DY11851	h^{+}	*atg18a-YFH::leu1+ his3-D1 leu1-32*

DY11884	h^+	*atg18a-YFH::leu1+ atg5-mCherry::natMX his3-D1 leu1-32*
DY11933	h^-	*atg5-mCherry::natMX his3-D1 leu1-32*
DY11886	h^+	*atg18aΔ::kanMX leu1-32::atg18a-YFH(leu1+) CFP-atg8::leu1+*
DY11890	h^+	*atg18aΔ::kanMX leu1-32::atg18a(FTTG)-YFH(leu1+) CFP-atg8::leu1+*
DY11855	*h?*	*atg5Δ::natMX leu1-32::tdh1-YFH(leu1+) cpy1-mCherry::kanMX CFP-atg8::leu1+*
DY11856	h^-	*leu1-32::tdh1-YFH(leu1+) cpy1-mCherry::hphMX CFP-atg8::leu1+*
DY11879	*h?*	*ctl1Δ::kanMX leu1-32::tdh1-YFH(leu1+) cpy1-mCherry::hphMX CFP-atg8::leu1+*
DY11926	*h?*	*ctl1Δ::kanMX atg17-YFH::leu1+ CFP-atg8::leu1+ leu1-32*
DY11853	*h?*	*ctl1Δ::kanMX atg18a-YFH::leu1+ CFP-atg8::leu1+ leu1-32*
DY11881	*h?*	*ctl1Δ::kanMX atg18b-YFH::leu1+ CFP-atg8::leu1+ leu1-32*
DY11885	*h?*	*ctl1Δ::kanMX atg1-YFH::leu1+ CFP-atg8::leu1+ leu1-32*
DY11852	*h?*	*ctl1Δ::kanMX atg2-YFH::leu1+ CFP-atg8::leu1+ leu1-32*
DY11887	*h?*	*ctl1Δ::kanMX atg5-YFH::leu1+ CFP-atg8::leu1+ leu1-32*
DY11888	*h?*	*ctl1Δ::kanMX atg6-YFH::leu1+ CFP-atg8::leu1+ leu1-32*
DY4132	*h?*	*ctl1Δ::kanMX atg14-YFH::leu1+ CFP-atg8::leu1+ leu1-32*
DY11910	*h?*	*atg9-mCherry::kanMX ctl1-YFH::leu1+ leu1-32*
DY11902	h^+	*atg9-mCherry::kanMX his3-D1 leu1-32*
DY3989	h^+	*ctl1-YFH::leu1+ his3-D1 leu1-32*
DY11906	h^-	*atg1Δ::kanMX atg9-YFP::kanMX CFP-atg8::leu1+ leu1-32*
DY11907	h^+	*atg2Δ::kanMX atg9-YFP::kanMX CFP-atg8::leu1+ leu1-32*
DY11897	*h?*	*atg9-YFP::kanMX zhf1-mCherry::kanMX CFP-atg8::leu1+ leu1-32*
DY11896	*h?*	*ctl1Δ::kanMX atg9-YFP::kanMX zhf1-mCherry::kanMX CFP-atg8::leu1+ leu1-32*
DY11908	h^+	*anp1-mCherry::ura4+ ctl1-YFH::leu1+ CFP-atg8::leu1+ leu1-32 ura4-D18 (or ura4+)*
DY11920	*h?*	*atg1Δ::kanMX anp1-mCherry::ura4+ ctl1-YFH::leu1+ CFP-atg8::leu1+ leu1-32 ura4-D18 (or ura4+)*
DY11911	*h?*	*atg2Δ::kanMX anp1-mCherry::ura4+ ctl1-YFH::leu1+ CFP-atg8::leu1+ leu1-32 ura4-D18 (or ura4+)*
DY11912	*h?*	*atg9Δ::kanMX anp1-mCherry::ura4+ ctl1-YFH::leu1+ CFP-atg8::leu1+ leu1-32 ura4-D18 (or ura4+)*
DY11895	*h?*	*ctl1Δ::kanMX atg9-YFP::kanMX atg17-mCherry::natMX CFP-atg8::leu1+ leu1-32*
DY11894	*h?*	*atg9-YFP::kanMX atg17-mCherry::natMX CFP-atg8::leu1+ leu1-32*
DY11937	h^-	*fsc1-YFH::leu1+ cpy1-mCherry::kanMX his3-D1 leu1-32*
DY11854	*h?*	*fsc1-YFH::leu1+ zhf1-mCherry::kanMX CFP-atg8::leu1+ leu1-32*
DY11918	*h?*	*atg1Δ::kanMX fsc1-YFH::leu1+ CFP-atg8::leu1+ leu1-32*
DY11917	*h?*	*atg11Δ::kanMX fsc1-YFH::leu1+ CFP-atg8::leu1+ leu1-32*
DY11919	*h?*	*atg13Δ::kanMX fsc1-YFH::leu1+ CFP-atg8::leu1+ leu1-32*
DY4001	*h?*	*fsc1-YFH::leu1+ CFP-atg8::leu1+ leu1-32*
DY11924	*h?*	*atg2Δ::kanMX fsc1Δ::natMX CFP-atg8::leu1+ leu1-32*

DY11921	*h?*	*atg5Δ::kanMX fsc1Δ::natMX CFP-atg8::leu1+ leu1-32*
DY11882	*h?*	*fsc1Δ::kanMX atg5Δ::kanMX leu1-32::tdh1-YFH(leu1+) cpy1-mCherry::hphMX CFP-atg8::leu1+*
DY11878	h^+	*fsc1Δ::kanMX leu1-32::tdh1-YFH(leu1+) cpy1-mCherry::natMX CFP-atg8::leu1+*
DY11922	*h?*	*fsc1Δ::kanMX leu1-32::cpy1-YFH(leu1+) his3-D1 ura4-D18*
LD259	h^+	*leu1-32 his3-D1 ura4-D18*
DY11923	*h?*	*aut12Δ::kanMX leu1-32::cpy1-YFH(leu1+) his3-D1 ura4-D18*
