# Supplementary material for: Global Analysis of Fission Yeast Mating Genes Reveals New Autophagy Factors
Source: PLoS Genet. 2013 Aug 8;9(8):e1003715. doi: 10.1371/journal.pgen.1003715 (PMC3738441; doi:10.1371/journal.pgen.1003715)
Supplement: Table S5 — The barcode sequencing data deposited at SRA. (PDF) [file pgen.1003715.s015.pdf]

Table S5

Sequencing data of the mating screens (SRA068523)

Run Name	Sameple Name	Uptag Index	Dntag Index
SRR770472	Input_0428_YES	CGAT	TATA
SRR770473	Input_0521_EMM	CGAT	TATA
SRR770474	Input_0521_YES	CGAT	TATA
SRR770476	Input_1108_YES	CGAT	TATA
SRR770477	Spore_0428_YES_SPA-45s	AGCT	TCGA
SRR770478	Spore_0428_YES_SPA-100s-30n	TAAT	AGGA
SRR770479	Spore_0428_YES_SPA-100s	CAGT	ATTA
SRR770480	Spore_0428_YES_SPA-200s	ATAT	CGTA
SRR770481	Spore_0521_EMM_EMM1g-45s	ATAT	CGTA
SRR770482	Spore_0521_EMM_EMM-45s	AGCT	TCGA
SRR770483	Spore_0521_EMM_EMM-225s	CAGT	ATTA
SRR770484	Spore_0521_EMM_EMMnoN-45s	TAAT	AGGA
SRR770485	Spore_0521_EMM_SPA-45s	ATC	GCG
SRR770486	Spore_0521_EMM_SPA-200s	CTA	GAT
SRR770487	Spore_0521_EMM_YEPD	TCA	GGC
SRR770488	Spore_0521_YES_EMM1g-45s	ATAT	CGTA
SRR770489	Spore_0521_YES_EMM-45s	AGCT	TCGA
SRR770490	Spore_0521_YES_EMM-225s	CAGT	ATTA
SRR770491	Spore_0521_YES_EMMnoN-45s	TAAT	AGGA
SRR770492	Spore_0521_YES_ME	GCC	TAG
SRR770493	Spore_0521_YES_SPA-45s	ATC	GCG
SRR770494	Spore_0521_YES_SPA-200s	CTA	GAT
SRR770495	Spore_0521_YES_YEPD	TCA	GGC
SRR770496	Spore_1108_YES_SPA-45s_20d	AGCT	TCGA
SRR770497	Spore_1108_YES_SPA-45s_25d	CAGT	ATTA
SRR770498	Spore_1108_YES_SPA-45s_30d	ATAT	CGTA
